# Whole Genome Sequencing Reveals Substantial Genetic Structure and Evidence of Local Adaptation in Alaskan Red King Crab

**DOI:** 10.1111/eva.70049

**Published:** 2024-12-31

**Authors:** Carl A. St. John, Laura E. Timm, Kristen M. Gruenthal, Wesley A. Larson

**Affiliations:** ^1^ Department of Natural Resources and the Environment Cornell University Ithaca New York USA; ^2^ National Oceanographic and Atmospheric Administration, National Marine Fisheries Service Alaska Fisheries Science Center, Auke Bay Laboratories Juneau Alaska USA; ^3^ College of Fisheries and Ocean Sciences, University of Alaska Fairbanks Fairbanks Alaska USA; ^4^ Alaska Department of Fish and Game, Division of Commercial Fisheries, Gene Conservation Laboratory Juneau Alaska USA

**Keywords:** local adaptation, population genomics, soft selective sweep, whole genome sequencing

## Abstract

High‐latitude ocean basins are the most productive on earth, supporting high diversity and biomass of economically and socially important species. A long tradition of responsible fisheries management has sustained these species for generations, but modern threats from climate change, habitat loss, and new fishing technologies threaten their ecosystems and the human communities that depend on them. Among these species, Alaska's most charismatic megafaunal invertebrate, the red king crab, faces all three of these threats and has declined substantially in many parts of its distribution. Managers have identified stock structure and local adaptation as crucial information to help understand biomass declines and how to potentially reverse them, with regulation and possible stock enhancement. We generated low‐coverage whole genome sequencing (lcWGS) data on red king crabs from five regions: The Aleutian Islands, eastern Bering Sea, northern Bering Sea, Gulf of Alaska, and Southeast Alaska. We used data from millions of genetic markers generated from lcWGS to build on previous studies of population structure in Alaska that used < 100 markers and to investigate local adaptation. We found each of the regions formed their own distinct genetic clusters, some containing subpopulation structure. Most notably, we found that the Gulf of Alaska and eastern Bering Sea were significantly differentiated, something that had not been previously documented. Inbreeding in each region was low and not a concern for fisheries management. We found genetic patterns consistent with local adaptation on several chromosomes and one particularly strong signal on chromosome 100. At this locus, the Gulf of Alaska harbors distinct genetic variation that could facilitate local adaptation to their environment. Our findings support the current practice of managing red king crab at a regional scale, and they strongly favor sourcing broodstock from the target population if stock enhancement is considered to avoid genetic mismatch.

## 1 Introduction

Fisheries are the largest mass‐harvested, wild food source on Earth. They help feed billions of people and support tens of millions of livelihoods (FAO 2016). Nearly two thirds of fisheries landings come from marine ecosystems, which are changing rapidly from anthropogenic influence (FAO 2016). Marine species in general face threats to their persistence from a multitude of recent ecological changes, including warming waters, ocean acidification, habitat destruction, marine pollution, and others. For commercially harvested species or species exposed to bycatch from commercial harvest, those threats are compounded by fishery mortality (Cheung et al. 2009; Swiney, Long, and Foy 2017). In particular, Arctic marine species are facing steady declines that are projected to continue into the foreseeable future (Szuwalski et al. 2020, 2023).

When species have declined to critical levels, managers and fishery rights holders seek new information to help recover stocks and/or more sustainably manage them. Increasingly, genetic tools are used to fill information gaps which support at‐risk species and the communities that depend on them (Taylor, Dussex, and van Heezik 2017). Genetic information is used to define management units, determine stock of origin, assess local adaptation, and genetically mark individuals, among other management‐relevant applications (Beacham et al. 2018; Moritz 1994; Ruzzante et al. 2019; Waples 1991). When applicable, genetic tools also provide insights into the magnitude of the genetic consequences associated with stock enhancement, which may include low fitness of stocked individuals, overfishing of wild populations versus stocked populations, reductions in genetic diversity, and inbreeding or outbreeding depression (Araki and Schmid 2010).

Next‐generation sequencing has now been used widely in fisheries for a decade, giving researchers greater power to differentiate among populations, define species boundaries, and detect adaptation to local environments (Benestan et al. 2015, 2016; Larson et al. 2014). Reduced‐representation sequencing tools have been instrumental for defining population units and assessing genetic diversity, but they are limited in their ability to detect local adaptation, which can occur at very narrow regions on the genome (Clucas et al. 2019; Larson et al. 2014; Lou et al. 2021; Tigano and Friesen 2016). Whole genome sequencing significantly enhances the detection of these narrow regions and can more accurately represent population structure and demographic histories at finer spatial and temporal scales. Unfortunately, it is often cost‐prohibitive for large sample sizes common in fisheries research. Low‐coverage whole genome sequencing (lcWGS) provides similar power as traditional whole genome sequencing at a cost comparable to reduced‐representation methods and likely represents a next step in genomics surveys of fisheries species (Lou et al. 2021).

We used lcWGS to examine the population structure and local adaptation in an iconic Alaskan commercial species, red king crab, *Paralithodes camtschaticus* (Tilesius, 1815). Red king crab management in Alaska could benefit from a better knowledge of population substructure and an enhanced understanding of local adaptation among populations. In the 1980s, Alaskan red king crab populations collapsed due to a combination of overharvest, bycatch, and climatic changes that began in the late 1970s (Bechtol and Kruse 2009; Dew and McConnaughey 2005). Nearly all populations then rebounded and together supported one of the most valuable commercial fisheries in the US (between $46 million and $146 million per year since 2010) (NOAA Fisheries 2019). However, Gulf of Alaska red king crab abundances never recovered, potentially due to skewed sex ratios toward females and sustained warmer water temperature associated with climate regime shifts in the 1970s and 1980s, and the fishery remains closed (Bechtol and Kruse 2009). Additionally, the Bristol Bay red king crab population, the largest remaining stock, was closed to fishing for 2 years in 2021 and 2022, prompting a request for a Federal Fishery Disaster recognition (Governor Dunleavy Requests Federal Fishery Disaster for Bristol Bay Red King Crab and Bering Sea Snow Crab Fisheries – Mike Dunleavy 2022; Westphal and Nichols 2022). These alarming declines, as well as the recent collapse of the snow crab fishery, another major crab fishery, has propelled research into population dynamics, basic biology and ecology, and stock enhancement programs for commercially fished Alaskan crab species and, specifically, red king crab (Long, Cummiskey, and Munk 2018; Long, Daly, and Cummiskey 2024).

Currently, red king crabs in the north Pacific Ocean are managed as six stocks: Bristol Bay, Pribilof Islands, Norton Sound, Aleutian Islands, Southeast Alaska, and Gulf of Alaska. This stock structure is based on inferences from oceanographic modeling of larval dispersal, the distribution of suitable settlement habitat, and the localized effects of fishing effort (Daly et al. 2020; Stevens 2014a). Fisheries geneticists have previously used genetic data from allozymes, targeted sequence capture of nuclear DNA, microsatellites, and mtDNA to help further resolve the stock structure, but they found evidence for only three genetic groupings in Alaska: Southeast Alaska, the Gulf of Alaska/eastern Bering Sea, and the Aleutian Islands/Norton Sound (Grant et al. 2011; Grant and Cheng 2012; Vulstek et al. 2013). These major groups are likely the result of isolation in separate glacial refugia prior to recolonization of their current habitat (Grant and Cheng 2012) and may not reflect recent demography of red king crab populations. SNPs and microsatellites revealed additional structure within Southeast Alaska (Grant and Cheng 2012; Vulstek et al. 2013), which is most likely the result of genetic drift occurring in small, relatively isolated populations. Other red king crab populations may also be isolated through strong self‐recruitment inferred from the modeling of ocean currents and vital rates (Daly et al. 2020).

In addition, local adaptation has not been explored in Alaskan red king crabs, though it is likely present given the species inhabits diverse environments from open oceanic shelves in the Bering Sea to small bays and fjords fed by glacial melt water in Southeast Alaska and the Gulf of Alaska (Grant, Zelenina, and Mugue 2014). Phenotypic differences across regions can be significant, although it is unclear whether these differences are due to phenotypic plasticity or whether they have a genetic basis. Some examples of region differences include the fact that populations from northern colder waters near Norton Sound mature at ~80% the size of populations from the warmer waters of Bristol Bay, but Norton Sound populations are 28%–38% more fecund (Otto, MacIntosh, and Cummiskey 1989). A better understanding of the mechanisms that influence life history differences could be especially important for a commercially harvested species like red king crab, which experiences fisheries mortality that varies over small scales (tens or hundreds of kilometers). Moreover, knowledge of local adaptation can help guide planning for stock enhancement, if implemented, to avoid stocking individuals maladapted to the targeted population.

Here, we generated lcWGS data for nearly 200 red king crabs in a study region spanning the Aleutian Islands, Norton Sound/Chukchi Sea, eastern Bering Sea, Gulf of Alaska, and Southeast Alaska. Our analysis of these data revealed undescribed population structure and evidence of local adaptation across Alaska. This information can inform spatially appropriate management and may help guard against overharvest of vulnerable stocks. Additionally, this information may guide potential enhancement programs toward minimizing negative impacts on important genetic variation and provide information on how adaptive differences among stocks may impact responses to specific environmental conditions. Finally, our findings will help inform fisheries management responses to population declines throughout Alaska and spatial shifts in distribution that may already be underway as the Bering Sea and North Pacific warm (Zacher, Kruse, and Hardy 2018).

## 2 Methods

### 2.1 Sample Collection and Laboratory Methods

**FIGURE 1:**
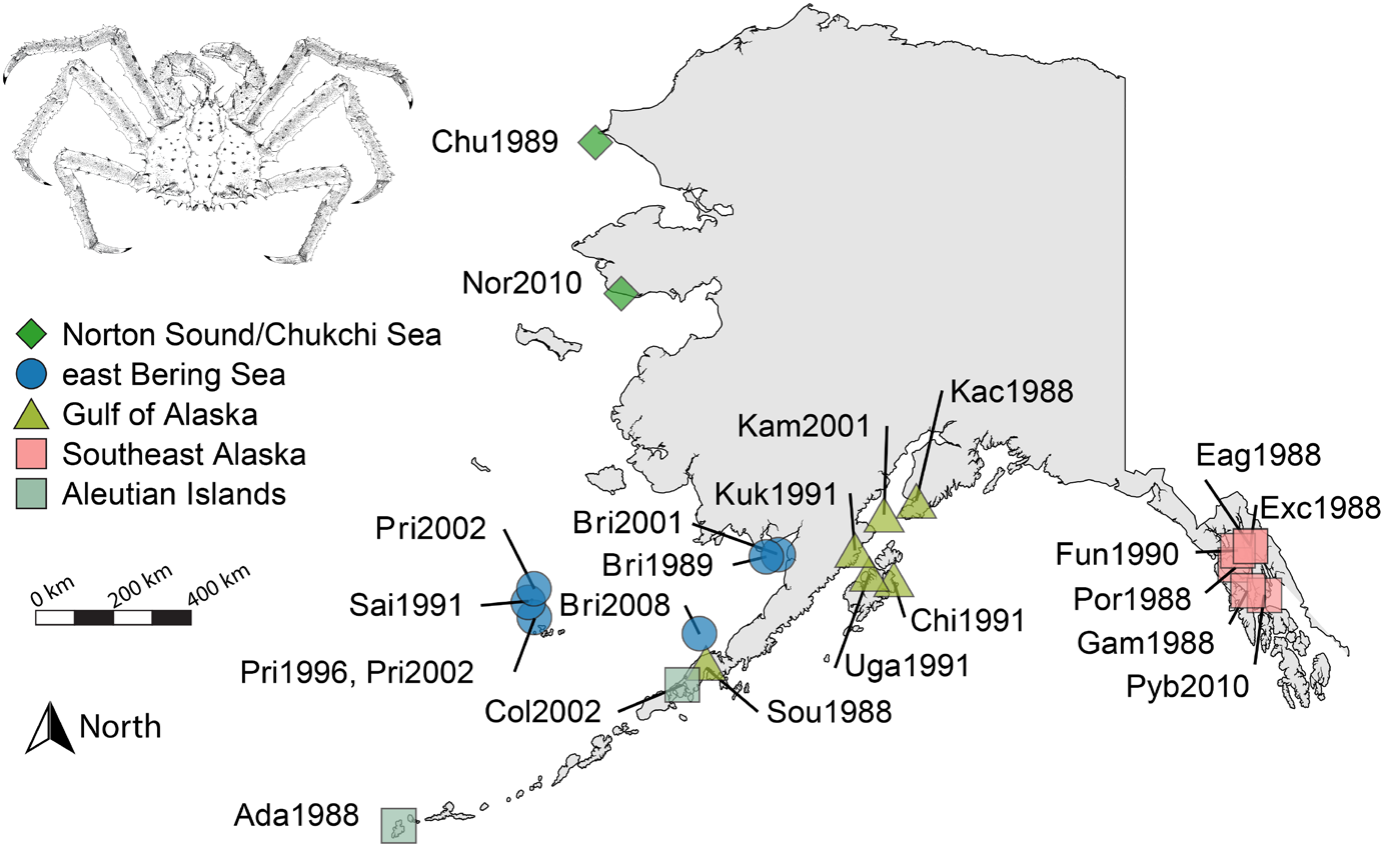
Map of collection sites and years of collections colored by regions: Aleutian Islands, eastern Bering Sea, Gulf of Alaska, northern Bering Sea, and Gulf of Alaska. Col2002 were likely caught further west in the Aleutian chain. Top left: Stipple drawing of a mature male Red King Crab.

Genetic material was collected from portions of muscle, heart, gill, and hepatopancreas from 192 adult red king crabs caught in National Oceanic and Atmospheric Administration (NOAA) and Alaska Department of Fish and Game fisheries surveys between 1988 and 2015. Samples collected before 1991 were frozen to −15°C and stored at −80°C, and samples collected after 1991 were immediately frozen in liquid nitrogen and then stored at −80°C until extraction. Over 95% of the samples (183/192) passed quality control filters (described below) and were included in this study (Table 1). Samples are from archives maintained by the Alaska Department of Fish and Game, and most of the samples included in this study were also included in Grant and Cheng (2012). DNA was extracted using a variety of methods but largely Qiagen DNAeasy blood and tissue kits (see Grant and Cheng 2012). Our sampling design prioritized samples from five major regions of interest to fisheries management: Norton Sound/Chukchi Sea (Nor/Chu), eastern Bering Sea (EBS), Gulf of Alaska (GOA), Southeast Alaska (SEAK), and the Aleutian Islands (AIs) (Figure 1). Collections from Cold Bay were likely caught further west in the Aleutian Islands based on experience from Alaskan red king crab biologists (Long W.C., personal comm.). Sexes were not noted for each sample, but sex ratios for all populations are similar, ranging between 1:1 (male:female) for GOA and 5:1 (male:female) for the Pribilof Islands. Survey catch data show that males and females are often caught at a ratio of 1:1 (male:female) (Hamazaki 2024; Palof 2023) regardless of the modeled abundance of sexes. Red king crab habitat varies with ontogeny. Post‐settlement and juvenile red king crabs require nearshore complex habitats, which then migrate to deeper, cooler offshore habitats that typically have soft bottoms (Stevens 2014a). Water temperature is generally the lowest in the Nor/Chu populations, slightly higher in EBS and AIs, and the highest in GOA (Lovrich 2014). Temperature associations in SEAK are not documented in the literature, but SEAK populations may have access to cold refuges in glacially fed fjords. Salinity is the highest for EBS and AIs populations, slightly lower for Nor/Chu, and the lowest salinity occurs for GOA and SEAK (Alaska Fisheries Science Center 2024; Johnson and Stabeno 2017; Oceanographic Station GAK1 Hydrographic Time Series from 1998 to 2020 2024).

**TABLE 1:** Geographic locations, sample sizes, and common site names of collection sites.

Region (abbreviation)	Collection site/Year	Latitude	Longitude	Sample size	Sample size per region
Norton Sound/Chukchi Sea (Nor/Chu)	Norton Sound 2010	64.5258	−165.7257	15	28
	Chukchi Sea 1989	68	−167	13	
East Bering Sea (EBS)	St. Paul Island 1991	57.178	−170.297	15	45
	Pribilof Islands 1996	56.82	−170	1	
	Pribilof Islands 2002A	56.82	−170	6	
	Pribilof Islands 2002B	57.5	−169.9833	7	
	Bristol Bay 2008	56.4519	−161.8869	4	
	Bristol Bay 2001	58.33	−158.07	6	
	Bristol Bay 1989	58.2	−158.6	6	
Gulf of Alaska (GOA)	Ugak Bay 1991	57.8547^a^	−153.5217^a^	5	44
	Chiniak Bay 1991	57.7	−152.4	7	
	Kachemak Bay 1988	59.63	−151.31	11	
	Kamishak Bay 2001	59.23283	−152.8203	8	
	Ugak Bay 2002	57.8547^a^	−153.5217^a^	3	
	South Peninsula 1988	55.518	−161.56	10	
Southeast Alaska (SEAK)	Port Frederick 1988	58.07	−135.57	5	35
	Excursion Inlet 1988	58.4203	−135.4439	6	
	Eagle River 1988	58.5259	−134.8216	5	
	Pybus Bay 2010	57.34387	−134.1312	11	
	Funter 1990	58.351	−134.8916	4	
	Gambier Bay 1988	57.45	−134.95	4	
Aleutian Islands (AIs)	Adak 2015	51.8627^a^	−176.6607^a^	7	16
	Cold Bay 2002	55.2073^a^	−162.7159^a^	3	
	Adak 1988	51.8627	−176.6607	6	

^a^ Latitudes and longitudes are approximate. Sample from Cold Bay likely originated from further west in the Aleutian Islands.

Library preparation for whole genome sequencing followed the methods of Baym et al. (2015) and Therkildsen and Palumbi (2017), modified by Euclide et al. (2023). Briefly, input DNA was normalized to 10 ng for each individual, and libraries were purified and normalized using SequalPrep plates (ThermoFisher Scientific, Waltham, MA, USA). Normalized pooled libraries were subject to a 0.6× size selection, purification, and volume concentration with AMPure XP from Beckman Coulter. Samples were sent to the University of Oregon Genomics and Cell Characterization Core Facility for whole genome sequencing using paired‐end 150 bp reads on an Illumina NovaSeq 6000 system with S4 chemistry. Forty‐eight individuals were multiplexed per lane to target a genome‐wide depth of coverage of 3× per individual given the estimated ~6 GB genome size of red king crab based on the blue king crab genome (Tang et al. 2021).

### 2.2 Quality Filtering, Obtaining Genotype Likelihoods

From raw demultiplexed fastq files, we first trimmed adapter sequences using Trimmomatic‐0.39 (Bolger, Lohse, and Usadel 2014; Lou and Therkildsen 2022). We then mapped filtered fastq files to the blue king crab ( *Paralithodes platypus* ) reference genome (GenBank accession: ASM1328300v1) using the bwa *mem* algorithm with the –M flag, which does not search for suboptimal hits (Li 2013; Tang et al. 2021). The resulting sam files were converted to bam files using samtools and indexed (Li et al. 2009). We removed PCR duplicates using picard v2.23.9 with the following settings: VALIDATION_STRINGENCY=SILENT REMOVE_DUPLICATES=true (*Picard Toolkit* 2019). We clipped overlapping paired reads using the bam clipOverlap program from the bamUtil repository (Jun et al. 2015).

SNPs were detected, and genotype likelihoods (GLs) were generated for those SNPs across all individuals using ANGSD‐0.933 (Korneliussen, Albrechtsen, and Nielsen 2014). We excluded regions with read mapping scores lower than 15 using –minMapQ 15, removed bases with quality scores < 20 using ‐minQ 20, and we excluded sites with a minor allele frequency (MAF) < 0.01. Minimum depth per locus was 183 and maximum depth was 3660. We also removed reads that mapped to multiple genomic regions (‐uniqueOnly 1), removed reads with a samtools flag above 255 (‐remove_bads 1), adjusted mapping quality scores for excessive mismatches (‐C 50), and only included reads with both mated pairs (‐only_proper_pairs 1). We generated a beagle‐formatted output file (‐GL 1), estimated minor allele frequencies (‐doMaf 1), and per‐population allele frequencies (doCounts ‐1) that were used for downstream analyses.

Finally, we removed SNPs with significant deviances in heterozygosity that may have arisen from erroneous mapping to paralogous locations on the genome using ngsParalog software (Linderoth, 2018). We first extracted a site list of all SNPs that passed the previous filters and generated a summary of sequencing depth for each SNP using samtools' mpileup tool, disregarding unmapped or duplicated reads (−ff UNMAP,DUP) and explicitly ignoring base and mapping quality scores (‐q 0 –Q 0). SNP‐wise coverage statistics were piped directly to ngsParalog to calculate the likelihood ratio of mismapping. Using a *χ* ^2^ test and Bonferroni correction, we identified and excluded SNPs with adjusted *p*‐values above 0.05. For site frequency spectra‐based analyses, we used the same options and filtering methods as described above but did not include a MAF filter and retained invariant sites.

### 2.3 Population Structure

The beagle‐formatted output from generating GLs was analyzed with principal component analysis using PCAngsd 0.99 (Meisner and Albrechtsen 2018) with the –pca flag, creating a covariance matrix across all individuals. Variable sample sizes can distort PCA space, so we randomly subsampled each region with *N* > 16 down to 16 individuals and reran the PCA using the same method on this subsampled dataset. We imported these covariance matrices into R for eigen decomposition and principal component analysis (PCA) visualization. We also subset the beagle file of GLs by geographic region (Nor/Chu, EBS, GOA, SEAK, and AIs) and ran PCAs for each region using the same method as used for the whole dataset.

We used NGSadmix to conduct admixture analysis with all samples (Skotte, Korneliussen, and Albrechtsen 2013). NGSAdmix was run with *K* = 1–8 populations and no minimum minor allele frequency filter (‐minMaf 0) with only polymorphic SNPs. Results were plotted using ggplot2 (Wickham et al. 2019). We assessed model fit using Akaike's Information Criterion with a correction for small sample size (AIC_c_) in R with a custom script.

### 2.4 Diversity and Differentiation Statistics

Since sample sizes for each specific collection were low (often < 10), we focused our analyses on five regions with robust sample sizes rather than on specific collections. These regions were: Nor/Chu (*N* = 28), EBS (*N* = 45), GOA (*N* = 44), SEAK (*N* = 35), and AIs (*N* = 16). Inbreeding coefficients were calculated per individual using ngsF at only polymorphic sites. We used ANOVA to test for differences in mean inbreeding among the five regions and a Tukey‐hsd test to determine significant differences between population pairs in R (R Core Team 2022). Diversity statistics including Watterson's theta and nucleotide diversity were calculated per region using folded site frequency spectra (SFS) in ANGSD using realSFS with the –fold 1 option (Korneliussen, Albrechtsen, and Nielsen 2014). We then calculated per position *F*_ST_ for each population pair using realSFS –whichFst 1 which specifies using the Hudson *F*_ST_ estimator (Bhatia et al. 2013). The Hudson estimator is more accurate when sample sizes differ among collections. We also used ANGSD to calculate weighted pairwise *F*_ST_ among the five genetic groups defined above.

### 2.5 Genome Scan for Highly Differentiated Genomic Regions

We focused our genome scan analyses on the five regional groupings from Table 1: Nor/Chu, EBS, GOA, SEAK, and AIs. To assess the patterns of genetic variation across the genome among these groupings, we first used a local PCA analysis with custom scripts following (Genomic‐Data‐Analysis/Scripts at Master · Therkildsen‐Lab/Genomic‐Data‐Analysis 2024; Li and Ralph 2019). Local PCA performs PCA on windowed sections of the genome, and comparisons between windows are made using an efficient simplification of Euclidean distance (*D*). We used 1000 bp windows in our analysis and used PCAngsd 1.10 (Meisner and Albrechtsen 2018) to run PCAs. Visualizations are produced by performing a multidimensional scaling (MDS) analysis on the dissimilarity matrix *D*, and we accomplished this in R using the cmdscale() function in the stats package (R Core Team 2022).

We then identified genomic regions displaying elevated differentiation between all possible pairwise combinations of populations using *F*_ST_ paired with a local score approach (Andrews et al. 2023; Fariello et al. 2017; Howe et al. 2024). First, we filtered out SNPs with a minor allele frequency < 0.05, a minimum mapping quality < 15, and a SNP *p* value > 10^−10^, and our depth filters were specific to each population (Table S1). Next, we generated allele count data for each SNP in each regional grouping using the –dumpCounts 3 flag in ANGSD. This step does not use individual identifiers, resulting in allele counts for each region, as opposed to each individual. Then, we ran a Fisher Exact Test (FET) at each SNP for each pairwise population comparison which generated a *p*‐value. Finally, we used the approach by Fariello et al. (2017) to identify outliers. This method uses a combination of SNP *p*‐values from the FET and proximity of statistically significant SNPs tuned by a smoothing parameter *ξ* = 2 to designate outlier regions. Significance thresholds were calculated for each chromosome (*α* = 0.01), and regions exceeding the threshold were considered outlier peaks. Only chromosomes with significant outlier peaks in at least one pairwise comparison between regions were plotted in Figure 4, as plotting all 104 chromosomes obstructed the visualization of outlier peaks.

We conducted additional analysis on chromosome 100 (chr 100), which contained the most conspicuous outlier region, with some of the highest *F*_ST_ values in the study, and had SNPs with elevated *F*_ST_ values in multiple pairwise comparisons. First, we generated a SNP heatmap for top outlier SNPs in this region to visualize the pattern of variation among SNPs. We generated allele dosages by polarizing genotype likelihoods of every individual by the mean GL of the major homozygous allele of the GOA population. At each SNP position, an individual's GLs are compared to the reference GLs. If all of the individual's GL values equal 0.33, the individual's dosage is assigned “NA.” If all GLs are not equal, then the individual is assigned the sum of the GL for the heterozygous genotype and 2 × the GL for the homozygous major allele of the reference GL. We then generated a linkage disequilibrium (LD) heatmap of the region of interest. To calculate LD, we used ngsLD which can account for uncertainty contained in genotype likelihood data (Fox et al. 2019). First, we prepared input files for ngsLD by subsampling a chr100 outlier beagle file for 1 in every 10 SNPs and removing the header and position information. We also prepared a position file by selecting the position information from the subsampled beagle file. Next, we ran ngsLD with a maximum distance of 100 kb. We plotted *R* ^2^ values from the ngsLD output using the LDheatmap package in R (Shin et al. 2006). We also generated a PCA using only the chr 100 outlier region using the same PCA methods described for the whole genome dataset.

We estimated Tajima's *D* for the GOA population across chromosome 100 (which contained the most notable outlier region) using the thetaStat program in ANGSD with a window size of 5000 and a step size of 1000. We compared mean Tajima's *D* values between the chr 100 outlier region and the remaining windows on chr 100 excluding the outlier region and tested for significant differences using a two‐tailed *t* test. The variances of each dataset were the same, but Tajima's *D* was not normally distributed in each dataset. Sample sizes were very high for both the chr 100 data and outlier region data, so we concluded that a *t* test would still provide an accurate approximation of significance between Tajima's *D* values.

Finally, we investigated the genes found in the identified outlier regions using information from gene annotation of the Blue King Crab genome from Tang et al. (2021) in the NCBI BLAST database (accession ASM3271660v1). All genes within each outlier region were documented, and their functions were investigated using discontiguous megablast of standard databases against the same eight species used by Tang et al. (2021): *Drosophila melanogaster* (Ensembl release 95), *Bicyclus anynana* (GCF_900239965.1), *Bombus terrestris* (GCF_000214255.1), *Stegodyphus mimosarum* (GCA_000611955.2), *Penaeus vannamei* (GCA_003789085.1), *Aedes aegypti* (GCF_002204515.2), *Mus musculus* (Ensembl), and *Mesobuthus martensii* (GCA_000484575.1). Only predicted genes that aligned to sequences isolated from the above species were recorded. Predicted genes with no alignments to searched databases on Genbank were not investigated further.

### 2.6 Mitochondrial Analysis

Our methods for analyzing mitochondrial genomes generally follow the data processing workflow described in Lou et al. (2018). Low‐coverage whole genome sequencing generates mitochondrial genome data at a high depth in addition to low‐coverage nuclear genome data. We aligned our reads to the red king crab mitochondrial genome assembly available on Genbank (JX944381.1) using the same alignment and filtering methods used to filter the nuclear dataset (Kim et al. 2013). We then estimated allele counts in ANGSD using the following flags: ‐doCounts 1 ‐minQ 20 ‐dumpCounts 4 ‐doBcf 1 ‐gl 1 ‐dopost 1 ‐domajorminor 1 ‐domaf 1. Depth counts were also calculated in ANGSD for quality control using these flags: ‐doCounts 1 ‐minQ 20 ‐dumpCounts 2. We then prepared data for haplotype analysis by converting the allele counts output from ANGSD to fasta files for each individual in our dataset. We filtered loci by rejecting those with a minimum depth < 4 and a minimum major allele frequency < 0.75 (Lou et al. 2018). We subset this fasta file in R by region and calculated haplotype diversity per population using the R package pegas (Paradis 2010). We also extracted, aligned, and analyzed a multiple sequence alignment of the cytochrome oxidase I (COI) gene to facilitate direct comparisons to past studies. Haplotype networks were generated using the pegas package in R. Pie charts showing the composition of different haplotypes per population were generated using the haploFreq() function in pegas and plotting with ggplot2 (Paradis 2010; Wickham et al. 2019).

## 3 Results

### 3.1 Sequencing QC, Depths, and Other Metrics

We sequenced 192 individuals and retained 173 after quality filtering. Nine of the individuals that did not pass quality control were excluded because of low‐quality sequencing, and 11 were excluded because of ambiguous metadata. The mean sequencing depth was 2.073×, and standard deviation was 0.637. After quality filtering by locus, we retained 8,973,301 SNPs for downstream analysis.

### 3.2 Genome‐Wide Patterns of Population Structure and Genetic Diversity

**FIGURE 2:**
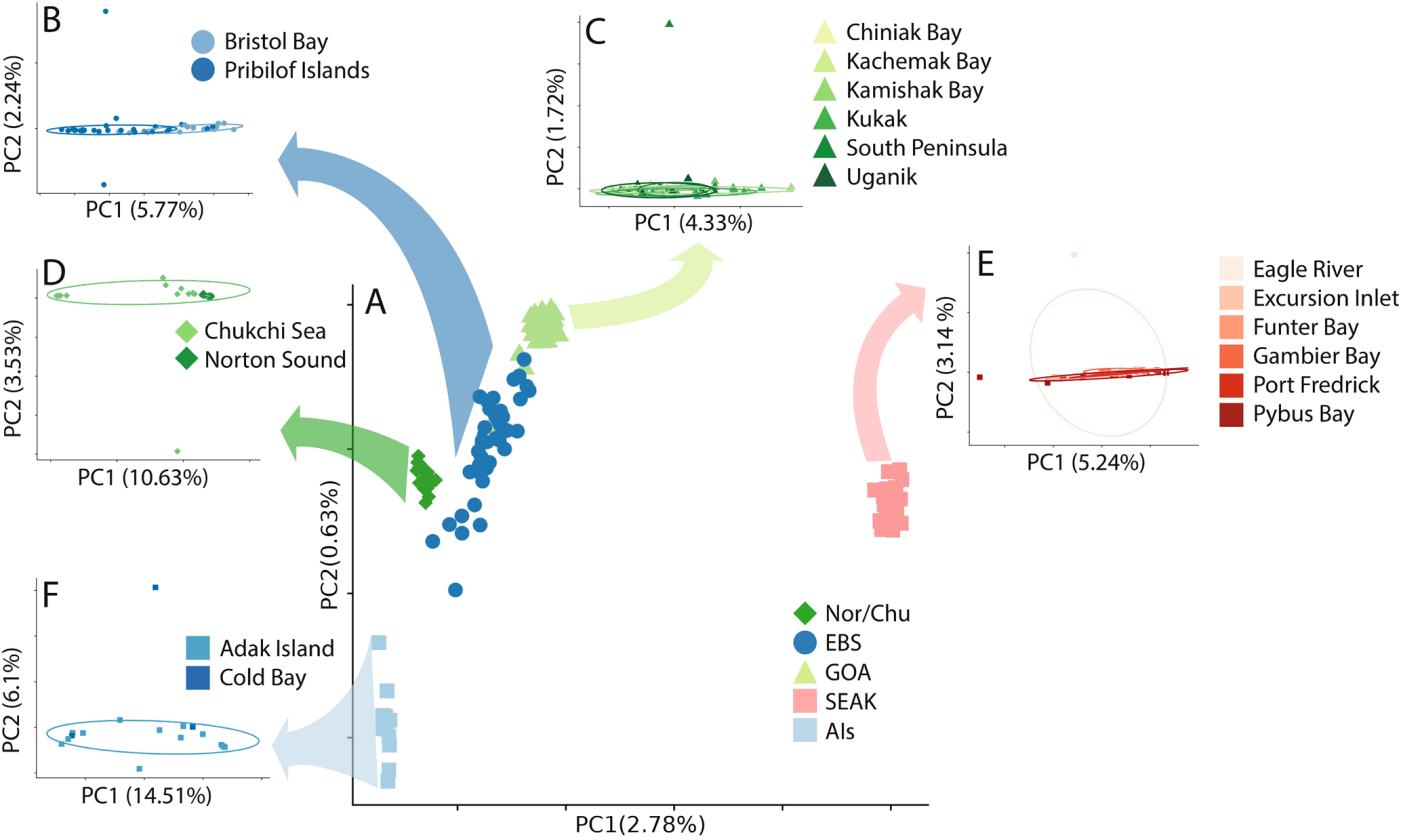
Principle component analysis. The central PCA projection includes all individuals colored by geographic region: Aleutian Islands (light blue squares), eastern Bering Sea (dark blue circles), Gulf of Alaska (light green triangles), Norton Sound and Chukchi Sea (dark green diamonds), and Southeast Alaska (pink squares). Arrows originating at each population point to independent PCA projections of each geographic region. The percent variation explained by each PC axis is printed next to the PC axis name. Ellipses in each regional PCA represent the 0.75 quantile of each population.

Principal component analysis revealed three major genetic groupings: (1) Southeast Alaska (SEAK); (2) Gulf of Alaska (GOA), eastern Bering Sea (EBS), and Norton Sound/Chukchi Sea (Nor/Chu); and (3) Aleutian Islands (AIs) (Figure 2). Genetic variation between SEAK and the other groups accounted for the majority of variation and was explained by PC1 (2.76% of variation explained). PC2 explains 0.63% of variation and primarily separates AIs from other groups.

Population structure varies widely within the three major genetic groups (Figure 2). Both SEAK and AIs populations appear homogenous with no obvious substructure (Figure 2E,F). However, small sample sizes in these groups prevent us from concluding that substructure does not exist. Conversely, we documented additional clusters within the GOA—EBS—Nor/Chu genetic group. Collections in this genetic group can be divided into three additional subgroups: (1) Nor/Chu which contains Norton Sound and the Chukchi Sea, (2) EBS, which contains collections from Bristol Bay and the Pribilof Islands, and (3) GOA, which contains collections from the south Alaska Peninsula to Cook Inlet. Hierarchical PCAs of subgroups revealed that Bristol Bay and Pribilof Island populations also may be separate genetic groups, although some Bristol Bay individuals grouped with the Pribilof Islands group and vice versa (Figure 2B). Hierarchical PCAs of other subgroupings did not show evidence of further structure (Figure 2B–F).

Admixture results generally agreed with the results from the PCA, but the PCA better detected subtle patterns of genetic structure (Figure S1). The most likely *K* was 1 or 2 using AIC_c_ (Figure S2), but our PCA and results from previous studies suggest that this value is not biologically reasonable (Grant and Cheng 2012; Seeb et al. 2002; Vulstek et al. 2013). With *K* = 3 ancestral populations—a more biologically reasonable value, admixture assigned all AIs individuals to group A and all SEAK individuals to group C, with little mixing (Figure S1). The other populations displayed mixed ancestries, but the proportions of those ancestries varied geographically. For example, the EBS and NBS contained more ancestry from the AI genetic group (admixture group B), whereas the GOA contained more ancestry from the SEAK genetic group (admixture group C). Admixture results with *K* = 4 were similar to *K* = 3, with one more genetic group found in the EBS and GOA collections but still no clear separation of these groups. At *K* = 5, NBS was largely its own genetic group, while the GOA and EBS were still somewhat mixed. Higher *K* values did not separate the GOA and EBS and resulted in higher AIC_c_ values (Figure S2).

Pairwise *F*_ST_ values among the five genetic groups were consistent with general trends shown by the PCA projection (Figure S3). The SEAK group had the highest *F*_ST_ values in pairwise comparisons to all other groups (*F*_ST_ ~ 0.01). SEAK was most differentiated from AIs and least differentiated from GOA (which is geographically proximate). AIs were least differentiated from Nor/Chu. The two least differentiated groups were GOA and EBS (*F*_ST_ = 0.00081).

**FIGURE 3:**
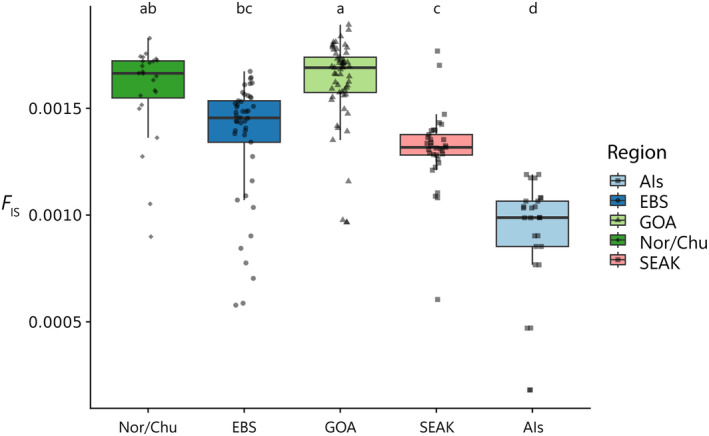
Inbreeding values for each region. Upper and lower hinges of the box are the 1st and 3rd quartiles. Whiskers extend to 1.5 interquartile range. Points are mean *F*_IS_ of individuals.

We documented a slight variation in the mean population inbreeding coefficients among groups (Figure 3). Most notably, GOA had a significantly higher (*p* < 0.01) mean inbreeding coefficient than the other populations except for Nor/Chu. Ais’ inbreeding values were significantly lower than any other population. Nucleotide diversity also varied among groups and was again the lowest in GOA, but no significant differences were found (Figure S4).

### 3.3 Genome Scan for Highly Differentiated Genomic Regions Putatively Under Selection

Local PCA (Figure S5) identified genomic regions where patterns of genetic variation differed from the genome‐wide signal. As expected, the MDS axis 1 of the local PCA identified loci that explained the genome‐wide pattern of population structure: Loci on this axis were distributed across the genome with no obvious outlier regions explaining a large portion of population structure. MDS axis 3 identified a region on chr 54 that, when analyzed using PCA, showed a possible pattern of reduced recombination on PC1 suggestive of a haploblock (11.09% variance explained) but with no relationship to population structure. MDS axes 4 and 5 identified similar regions that showed haploblock patterns on chromosomes 4 and 54. PCAs of these outlier regions separated out by haploblock allele on PC1 and by population structure on PC2. MDS axis 7 identified another region on chr100 than may correspond with a haploblock, but the pattern was much less clear than the above examples. None of the putative haploblocks identified by local PCA overlapped with *F*_ST_ outlier regions identified by local score, nor did they correlate with the population structure or geographic location.

**FIGURE 4:**
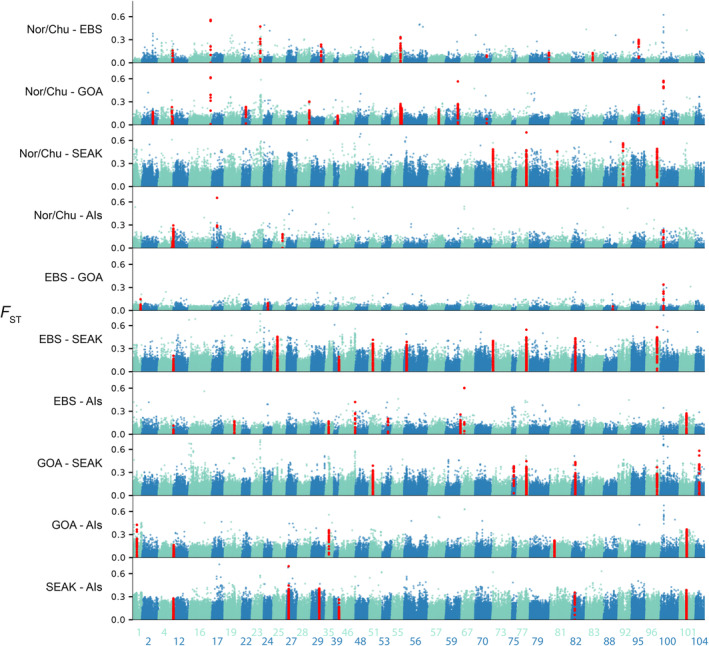
*F*_st_ genome scans of all pairwise comparisons of the five populations: Aleutian Islands, eastern Bering Sea, Gulf of Alaska, northern Bering Sea, and Southeast Alaska. *F*_st_ is on the *y*‐axis and chromosome and position are on the *x*‐axis. Chromosomes are numbered on the *x*‐axis. Note that only chromosomes with outlier regions identified by local score are plotted. Points represent SNPs. Colors of points alternate by chromosome to distinguish between chromosomes. Outlier regions identified by local score are colored red.

Outlier regions potentially associated with divergent selection were identified with analyses of genetic differentiation and local score. For local score analysis, all 10 possible pairwise comparisons (excluding self‐comparison) were made among the five populations. This approach identified 51 *F*_ST_ outlier regions distributed across 40 chromosomes (Figure 4; only chromosomes with significant outliers are shown; Table S2, list of significant regions). Chromosomes 12 and 55 contained the most outlier regions with three, seven chromosomes contained 2 outliers, and the remaining 31 chromosomes each contained one outlier. Nor/Chu had the most outlier regions in pairwise comparisons (35 regions), followed by GOA and EBS (31 regions each), AIs (23 regions), and lastly SEAK (21 regions).

**FIGURE 5:**
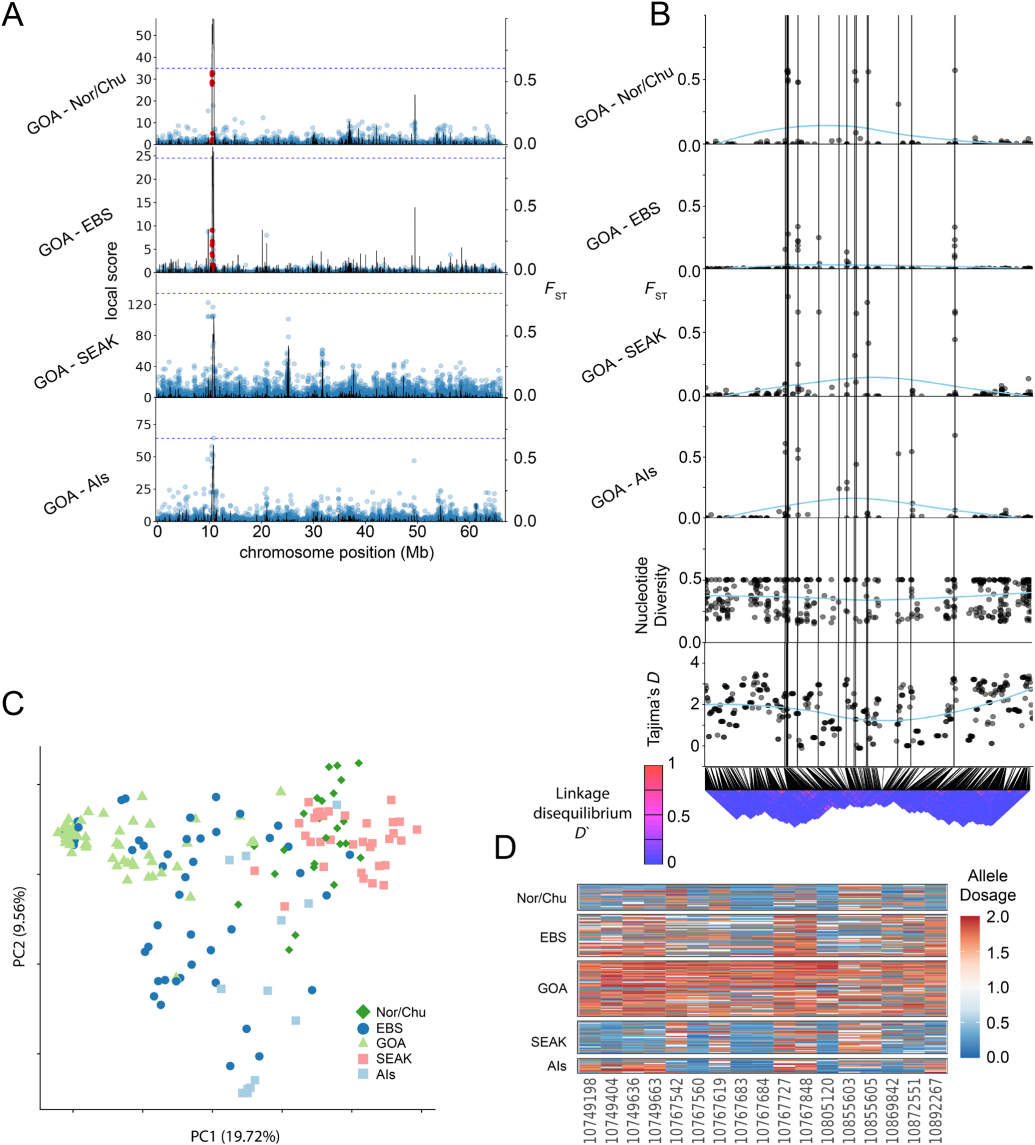
Localized *F*_ST_ peaks suggest local adaptation. (A) *F*_ST_ scan of chr 100 with position on the *x* axis and *F*_ST_ and local score on the right and left *y*‐axes respectively. Each dot is the *F*_ST_ value at each SNP and RED dots indicate outlier SNPs identified by local score. The black line in each plot is the local score value at that SNP. The dotted line is the local score significance threshold calculated for each population comparison. Not that the local score *y* axis changes in each plot. (B) Zoomed in *F*_ST_ scans of the outlier regions from chr 100. The *x*‐axis is physical position on chr 100 and is the same for each plot. The blue line shows mean *F*_ST_ fitted using a local polynomial regression from geom_smooth() in the R package ggplot2. Vertical lines mark outliers identified using local score. Nucleotide diversity, Tajima's *D*, and linkage disequilibrium (LD) are also plotted on the same scaled *x*‐axis below the *F*_ST_ plots. SNPs in the LD heat map are plotted adjacent to each (not based on physical distance) but lines above the LD heat map point to their physical position on the chromosome. (C) PCA of just the outlier loci plotted in panel C. Principal component (PC) 1 is plotted on the *x*‐axis and PC 2 is plotted on the *y*‐axis. (D) SNP heatmap at outlier loci. On the *x*‐axis are loci corresponding to the vertical lines on zoomed *F*_ST_ plots, and individuals are plotted on the *y*‐axis grouped by region. Alleles were polarized by the mean allele dosage of all Gulf of Alaska individuals. More blue colors are less similar to that Gulf of Alaska individual and redder colors are more similar to that individual. Values near 2.0 and 0.0 represent likely homozygosity and values near 1.0 represent likely heterozygosity. Gray represents missing data.

Notably, the chr 100 region consistently contained some of the highest *F*_ST_ values in the study, prompting us to investigate further (Figure 4). SNPs in the chr 100 region frequently reached *F*_ST_ values > 0.5 in pairwise comparisons and up to 0.8 between GOA and SEAK (Figure 5A). The high *F*_ST_ SNPs are grouped in several clusters within a ~ 300 kb candidate window between positions 10,744,898 and 11,050,174, rather than a single island of differentiation, suggesting several loci in the region may be under selection (Figure 5B). Linkage disequilibrium is slightly higher in the chr 100 candidate region compared with adjacent regions, but a large, conserved LD block does not exist (Figure 5B). Additionally, no obvious pattern of reduced nucleotide diversity is present in this region, but Tajima's *D* was slightly locally depressed across the region (Figure 5B). Tajima's *D* reached negative values at six windows in the outlier region, but mean Tajima's *D* values across this region were positive, 1.45. Tajima's *D* was 0.44 lower (*p* = 2.32 × 10^−5^, 95% CI = ±0.39) than the chr 100 mean Tajima's *D* values of 1.89.

PCA of the chr 100 outlier region (Figure 5C) revealed three discrete clusters corresponding to (1) GOA, (2) Nor/Chu and SEAK, and (3) AIs. PC1 separates the GOA from Nor/Chu, SEAK, and AIs clusters and PC2 separates AIs from the Nor/Chu and SEAK cluster and GOA. Interestingly, Nor/Chu and SEAK group together despite being the most differentiated in the genome‐wide PCA (Figure 2). EBS is scattered across the PCA space and slightly shifted away from Nor/Chu and SEAK clusters. A heatmap of only the SNPs above local score significance thresholds assisted with the interpretation of the chr 100 outlier PCA projection. The heatmap showed that GOA genotypes are generally different from Nor/Chu, SEAK, and AI genotypes (Figure 5D). However, a number of SNPs do not follow this pattern and instead differentiate comparisons such as AIs versus SEAK or EBS versus GOA (Figure 5D).

We found 26 predicted genes in the blue king crab genome annotation falling within all outlier regions identified by the local score. When blasted against other invertebrate annotated genomes, nine of the predicted genes aligned with glutamate‐gated chloride channel variants, and one gene aligned with a ribosomal protein S4; however, the e‐values of hits were relatively high (between 10^−5^ and 10^−40^). The remaining predicted genes did not align with any gene in Genbank (Table S3).

### 3.4 Mitochondrial Analysis

**FIGURE 6:**
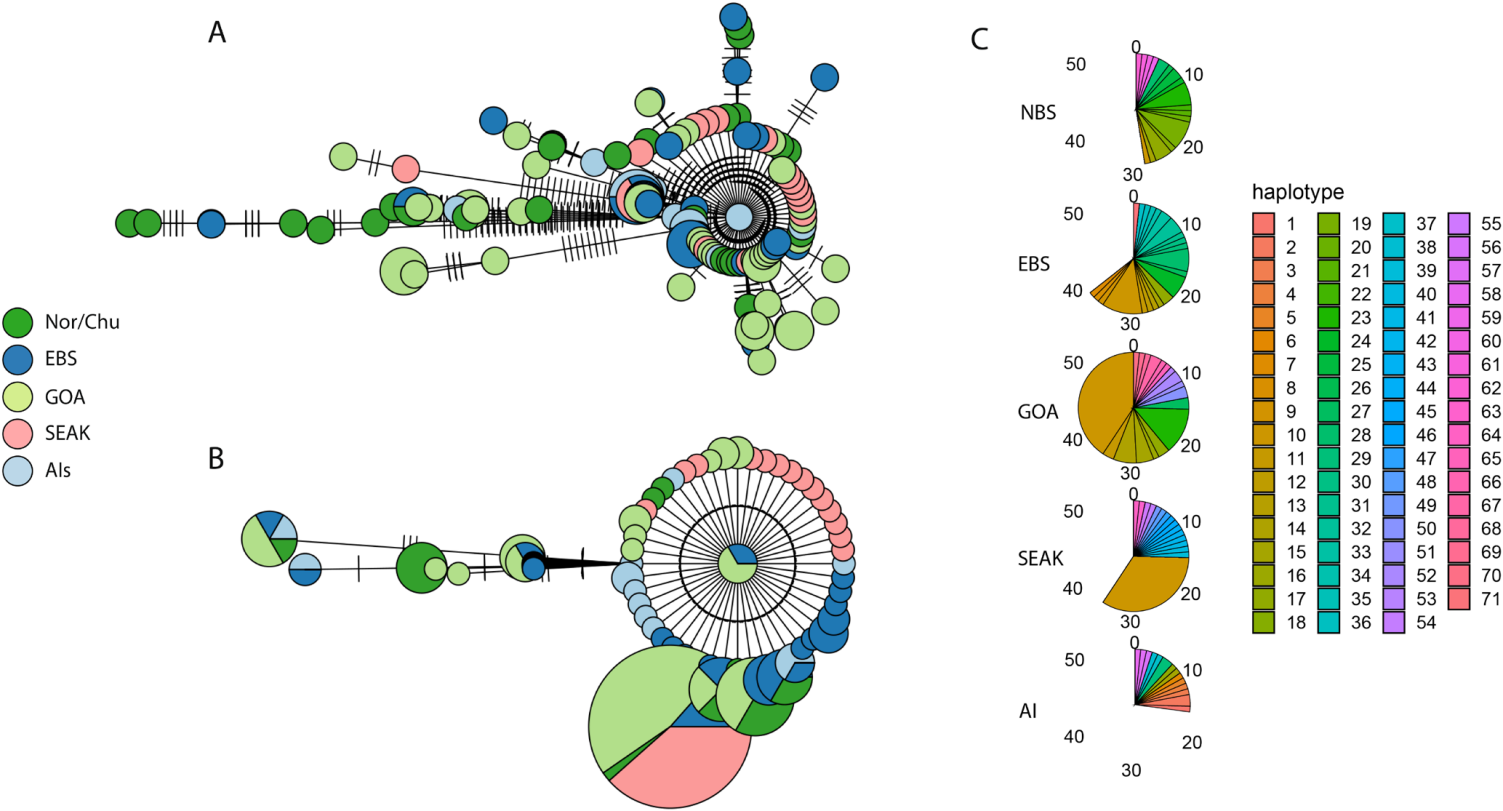
Haplotype networks and per‐population frequencies from mitochondrial data from all individuals. The top network (A) uses the entire mitochondrial genome and the bottom network (B) uses just COI. The size of pie graphs is scaled by number of individuals with that haplotype. Each tick on lines connecting haplotype pie graphs represents 1 bp difference. The colors representing geographic regions are consistent for each network. (C) Frequency of each COI haplotype in each population represented as pie charts.

Haplotype diversity was nearly identical across all populations, ranging from 0.99 to 1.00. Haplotype networks for the whole mitogenome (Figure 6A) and COI (Figure 6B) differed substantially, but individuals did not group by population in either network. In the full mitochondrial genome, the majority of individuals had unique haplotypes, and remaining individuals shared haplotypes with only two or three other individuals (Figure 6A–C). Shared haplotypes mostly included individuals from the same populations, but some shared haplotypes included a mix of populations. SEAK Alaska individuals were found in only one shared haplotype, with the rest having unique haplotypes. Using only COI (Figure 6B), we found one primary haplotype (haplotype 10 in Figure 6C) that included individuals from all populations except AIs. This haplotype may also be present in Ais, but the sample size in that region was small (*N* = 16). We found the most haplotype sharing in GOA where 24 out of 59 GOA individuals had haplotype 10 and eight had haplotype 23, with other haplotypes shared among four or fewer individuals. Beyond the primary haplotype, we found ~10 additional haplotypes shared by two or more individuals. SEAK individuals without the primary haplotype each had unique haplotypes. Ten haplotypes differed by four or more base pairs from the primary haplotype and included individuals from all populations except SEAK. In general, the only regional trends that we observed were generally lower diversity in SEAK and GOA and more singleton haplotypes in SEAK (Figure 6C).

## 4 Discussion

Analysis of whole genome data revealed substantial structure in Alaskan red king crab which is likely the result of recolonization from different glacial refugia and, more recently, selection and genetic drift. Population structure was observed across as little as a few hundred kilometers, which represents much finer scale structure than expected for a marine invertebrate with pelagic free‐swimming larvae based on oceanographic data (Kinlan and Gaines 2003; Shanks, Grantham, and Carr 2003, but see Benestan et al. 2015). Genetic studies of nearshore species have found similarly fine‐scale population structure to our study (Coscia et al. 2020; Kelly and Palumbi 2010; Morales‐González et al. 2019; Xuereb et al. 2018), but only Benestan et al. (2015) found comparable structure in an offshore spawning species such as red king crab. Our results indicate that Alaskan red king crab populations should be managed on scales that take into consideration these boundaries to ensure that the rich portfolio of genetic diversity found in red king crabs is maintained. Additionally, we documented multiple regions of the genome displaying high genetic differentiation consistent with divergent selection, including an extremely diverged region on chr 100. If stock enhancement becomes a management tool, caution should be exercised to ensure that captive breeding and stocking does not introduce alleles that may erode population structure and local adaptation.

### 4.1 Genetic Diversity

GOA and Nor/Chu had the highest inbreeding values and lowest nucleotide diversities of any population measured in this study. This finding contrasts with microsatellite and mitochondrial DNA results from Grant and Cheng (2012) and Vulstek et al. (2013) who instead found that SEAK had the lowest genetic diversity. However, Grant and Cheng (2012) also failed to detect reductions in diversity in SEAK with nuclear SNPs. Despite GOA and Nor/Chu having the highest inbreeding values among Alaskan red king crab populations studied here, their inbreeding values of 0.00162 and 0.00154, respectively, are still extremely low, well below other studies of imperiled populations (> 0.1) (de Jager et al. 2021; Mueller et al. 2022), indicating that this population does not carry elevated genetic load. Though the GOA red king crab fishery collapsed and has not recovered to fishable abundance, it seems this population has not experienced measurable negative genetic health consequences as a result. This is not entirely surprising as research, even in extreme fishery collapses, finds that genetic impacts occur slowly and may not manifest at all despite intense fishing (Pinsky et al. 2021). It is unclear why Nor/Chu individuals display higher inbreeding values than AIs, EBS, and SEAK, but possible explanations include smaller population size due to fewer food resources, limited settlement habitat, or a recent bottleneck.

### 4.2 Population Structure

We identified three major genetic groups (SEAK, GOA—EBS—Nor/Chu, and AIs), similar to previous genetic studies based on mitochondrial DNA, microsatellites, and nuclear SNPs (Grant, Zelenina, and Mugue 2014; Grant and Cheng 2012; Vulstek et al. 2013), though these studies grouped Norton Sound with AIs instead of with EBS and GOA. Also similar to prior work, we found the SEAK genetic group to be the most genetically distinct (*F*_ST_ range between 0.00839 and 0.01335 in pairwise comparisons), with less but still substantial amounts of structure between the remaining two groups. Whole genome sequencing made it possible to identify additional substructures within the three major genetic groups. We found that PCA clearly separates Nor/Chu (*F*_ST_ = 0.00118–0.01114), EBS (*F*_ST_ = 0.00081–0.0.00998), and GOA (*F*_ST_ = 0.00081–0.00839), which are all managed separately but not previously identified as unique groups.

In contrast to previous studies, we found potential substructures within EBS and Nor/Chu. Individuals from Bristol Bay and the Pribilof Islands (both EBS populations) grouped separately using PCA, though some individuals fell within the reciprocal grouping in the PCA space. We interpret this separation as two distinct populations that may occasionally exchange migrants. In Nor/Chu, Norton Sound individuals grouped tightly, separate from the Chukchi Sea, which were more dispersed in our PCA projection (Figure 2D). Our sample sizes are too small for Norton Sound and Chukchi Sea to make concrete inferences about the population structure in these regions, but the pattern we observed suggests more sampling in the northern Bering Sea and Arctic Ocean may reveal additional population structures. Substructures within larger groups may bias estimates of diversity via the Wahlund Effect (Wahlund 1928) or other mechanisms (Chikhi et al. 2010) that may falsely estimate pairwise and expected genetic differences. We found no evidence for substructures in most of our grouped regions (GOA, SEAK, and AIs), and the substructure in EBS and NBS was subtle and is unlikely to affect our measures of diversity. Had substructure in EBS and NBS affected diversity estimates, we might have expected consistent high or low estimates of diversity in these regions, but this was not the case (Figure 3). Finally, we gathered samples from collections dating between 1988 and 2015, which could introduce temporal bias into our population genetic analysis. However, this timespan only represents 3–4 generations for red king crab as they mature at 6–8 years of age (Stevens 2014b). Exploratory analyses using PCA to assess temporal population structure unsurprisingly found no grouping of samples by year on all PCs tested.

AIC of admixture results found *K* = 1 was the most likely model, which likely reflects that fact that the structure is still relatively low in our system compared to other organisms (i.e., *F*_ST_s less than or near 0.01). In general, admixture analyses were not able to differentiate genetic groups as clearly as PCA. Despite this, admixture analysis at higher *K* values appeared to produce biologically relevant results and demonstrated that AIs and SEAK populations had the most readily separable ancestry, followed by NBS, whereas EBS and GOA populations were more genetically similar. In particular, *K* values of 3–5 produced clustering patterns that generally matched results from the PCA.

Our analysis of mitochondrial data indicated proportionally fewer haplotypes in SEAK and GOA populations, which is concordant with Grant and Cheng (2012), but the haplotype diversity metric was nearly the same for all populations (between 0.99 and 1.00). These values are high, which is likely the result of large population sizes in each region. High haplotype diversity is consistent with the low inbreeding coefficient values we found using nuclear DNA. Unlike Grant and Cheng (2012), we were unable to detect any population structure in mitochondrial data across all populations. This was likely because we sampled 183 individuals compared to the 1278 sampled by Grant and Cheng (2012), which allowed them to accurately characterize the frequencies of over 80 haplotypes. Nevertheless, our analysis of the full mitochondrial genome revealed some patterns that were not evident in Grant and Cheng (2012): most notably, we found that some shared haplotypes (haplotypes shared by two or more individuals) contained individuals from SEAK and that SEAK was therefore not solely represented exclusively by individuals with the most common haplotype or singleton haplotypes, which was not evident from COI data. We thus suggest incorporating mitochondrial analysis into lcWGS studies as standard practice because this analysis is simple and provides added value for population genetic studies including facilitating comparisons to previous mtDNA work (Lou et al. 2018).

While the spatial extent of our study is similar to the two most relevant previous studies of king crab population structure (Grant and Cheng 2012; Vulstek et al. 2013), our study design differs substantially. Sample sizes for each collection in previous studies were generally near 50, whereas ours were ~10. However, we genotyped orders of magnitude more nuclear markers and were able to investigate variation across the full mitochondrial genome rather than a portion of a single gene. This increase in marker number, density, and distribution across the genome facilitated identification of important, previously unrecognized population structure, most notably between GOA and EBS. However, we also failed to identify the fine‐scale structure in SEAK documented previously. These observations provide a good reminder that lower coverage whole genome sequencing requires robust sample sizes when investigating fine‐scale genetic variation, even more so than high depth methods (Lou et al. 2021).

Population genetic data for marine species in Alaska are generally lacking, but some data are available to help place our results in a broader context. Detectable genetic population structure for marine species in Alaska often appears to be driven by genetic differentiation that occurred when populations were isolated in glacial refugia prior to post‐glacial recolonization. For example, Pacific herring ( *Clupea pallasii* ) displays a large genetic break near Kodiak consistent with colonization from separate glacial refugia (Liu et al. 2011), and chum salmon ( *Oncorhynchus keta* ) shows a similar genetic break on the northern Alaska Peninsula (Petrou et al. 2013). In other cases, divergence from glacial refugia is lacking, either due to connectivity through the last glacial maximum or from secondary contact and homogenization of different glacial refuge lineages, in each case indicating generally high connectivity. For example, Jasonowicz et al. (2017) and Timm et al. (2024) did not document any structure in sablefish ( *Anoplopoma fimbria* ) using RADseq and whole genome data (respectively); very subtle genetic structure was documented in walleye pollock ( *Gadus chalcogrammus* ) based on microsatellites (O'Reilly et al. 2004), and no structure was documented in snow crab ( *Chionoecetes opilio* ) from the Bering, Chukchi, and Beaufort seas based on microsatellites (Albrecht et al. 2014).

It is likely that unique aspects of red king crab life history have led to the much higher levels of genetic structure than typically seen for marine crustaceans in Alaska (Albrecht et al. 2014; Johnson 2019; Siddon and Grant 2018). The pelagic larval duration for red king crab is long (2–3 months), providing ample opportunity for long distance movements (Stevens 2014a) (but see Shanks (2009) for overestimation of larval dispersal). However, red king crabs require complex benthic habitat to successfully settle out of the planktonic phase, and those complex habitats have a patchy distribution across Alaska (Daly et al. 2020; Stevens 2014a). Distances between suitable settlement habitats may make connectivity between populations unlikely, despite large potential for dispersal. Substrate type, epibenthic faunal communities, and physical factors like temperature and salinity differ throughout the range of red king crab in Alaska (Stevens 2014a; Zheng and Kruse 2006), potentially creating strong selective gradients at the crucial postlarval settlement stage. Adaptation to local temperature (Cure et al. 2017) and growth conditions (Jørgensen et al. 2020) at the postlarval settlement stage has been observed in other marine species despite high dispersal potential and may be at least partially responsible for the genetic structuring observed in our study.

### 4.3 Evidence of Local Adaptation

Outlier regions identified with local PCA suggested several putative haploblocks that could represent structural variants such as inversions, but none of them correlated with the population structure or overlapped with *F*_ST_ outlier loci. We suspect these haploblocks are the result of neutral processes given no obvious associations with the population structure or candidate loci. Although structural variants such as inversions are often linked to local adaptation between populations with a high gene flow (Akopyan et al. 2022; Euclide et al. 2023; Schaal, Haller, and Lotterhos 2022; Tigano and Friesen 2016), they have also been found to be putatively neutral in other marine species, including Atlantic Halibut (Kess et al. 2021) and sablefish (Timm et al. 2024). We mapped sequence reads to the Blue King Crab genome, which influences the reference orientation of putative haploblocks, but our inferences regarding their relative location and interaction with candidate loci or population structure are robust to differences in reference orientation. A chromosome‐level assembly of a red king crab genome accompanied by long‐read sequencing of individuals with each haplotype of a putative haploblock would help determine if the haploblocks found here are inversions or another genomic structure that reduces recombination.

Genome scans using a local score method identified 22 outlier regions on 21 chromosomes. Approximately half of the regions were identified in single pairwise population comparisons and about a quarter were identified in three or more pairwise comparisons. All population comparisons contained at least two outlier regions, suggesting that at least some degree of local adaptation is occurring in all populations included in our study. As discussed previously, biotic and abiotic variables differ substantially among the populations in our study and could lead to highly variable selective pressures. We hypothesize that the outlier regions we identified reflect local adaptation to the highly variable habitats that red king crab rely on at early life stages. Outlier regions contained nine predicted glutamate‐gated chloride channel variants, which perform many general cellular functions in arthropods and other invertebrates (Wolstenholme 2012). The breadth of functions that glutamate‐gated chloride channels perform in invertebrates is too large to identify any one function that may be involved in local adaptation. Had we analyzed phenotypes along with genotypes in this analysis, we may have been able to better identify a function associated with glutamate‐gated chloride channels that may be under selection. Additionally one ribosomal protein subunit S4 analogue associated with cellular processes like hypoxia responses (Ding et al. 2022) was found in the chr100 outlier region.

We identified the chr 100 outlier region as particularly notable because of its extremely high genetic differentiation (*F*_ST_) in multiple pairwise comparisons. The largest divergence at this region was between GOA and SEAK, but nearly all population pairs showed elevated differentiation at a few SNPs at a minimum in this region. LD is slightly elevated between SNPs in the region but does not form a uniformly high LD block, as would be expected in a hard selective sweep or structural variant such as an inversion. Similarly, we did not observe a drop in nucleotide diversity that would suggest a hard selective sweep, but Tajima's *D* was significantly lower than the chr 100 mean value providing additional evidence of selection on this region. Further examination of genotype frequencies at SNPs in the region revealed complex patterns of divergence. Most of the highly differentiated SNPs are diverged between the GOA and other populations, but there are a number of SNPs that differentiate specific population pairs. For example, there are SNPs that differentiate the AIs from SEAK and Nor/Chu and SNPs that differentiate EBS from GOA. These patterns hint at complex signatures of selection.

Taken together, our results suggest that the putative adaptive divergence on chr 100 likely arose through a soft selective sweep (Berg and Coop 2015). Beneficial alleles derive from multiple individuals of a large population (*N* > 10^5^) in soft sweeps; therefore, the genetic signatures of soft sweeps include high haplotypic variation, minor or absent reduction in Tajima's *D*, and inconsistent LD patterns as observed in our data (Figure 5B) (Hermisson and Pennings 2017; Jensen 2014; Messer and Petrov 2013). A soft sweep is biologically plausible as red king crab populations are larger than 10^6^ individuals (Palof and Siddeek 2022), and their background genetic variation is more likely to harbor variants involved in local adaptation than smaller, more isolated populations (Hermisson and Pennings 2017; Messer and Petrov 2013). Several ecological patterns across the Alaskan distribution of red king crabs could generate a soft selective sweep for a particular genotype and phenotype. Year‐round average sea surface temperature varies between the Gulf of Alaska, the warmest region in this study (Litzow et al. 2020; Sea Surface Temperature | National Marine Ecosystem Status 2023), and the coldest regions, Norton Sound/Chukchi Sea and glacially fed fjords of Southeast Alaska. Nor/Chu red king crabs mature earlier and are more fecund than crab from the warmer waters inhabited by EBS populations: Potentially an adaptation to colder year‐round temperatures (Otto, MacIntosh, and Cummiskey 1989). SEAK populations may experience year‐round colder temperatures when they occur in glacially fed fjords. However, without phenotype data, we are unable to definitively associate genetic variation in the chr 100 region with a warm‐adapted phenotype. Still, variation in the chr 100 region found in GOA suggests that it may harbor some locally adapted alleles that are differentiated from all other Alaskan populations, particularly AIs, Nor/Chu, and SEAK. Preservation of this unique diversity should be considered in recovery efforts of the Gulf of Alaska red king crab fishery. Future studies could focus on resampling populations of interest and include phenotype data paired with a genotyping‐in‐thousands by sequencing (GT‐seq) approach targeting outlier regions to further describe local adaption while minimizing sequencing costs (Campbell, Harmon, and Narum 2015).

### 4.4 Management Implications, Conclusions, and Future Directions

Our broader sampling of the genome, as compared to previous genetic studies of Alaskan red king crab, documented six genetically distinct populations in Alaska: Southeast (SEAK), Gulf of Alaska (GOA), Bristol Bay, Pribilof Islands, Aleutian Islands (AIs), and Norton Sound/Chukchi Sea (Nor/Chu). Populations vary substantially in their genetic similarity. SEAK is most genetically distinct, and GOA, Pribilof Islands, and Bristol Bay are most genetically similar. We found genomic patterns suggesting local adaptation among all populations of red king crab, and a specific region on chr 100 suggesting GOA (with some overlap with EBS) harbors unique locally adapted alleles. Red king crabs live in environments with different temperatures, salinities, and hydrological regimes throughout Alaska, and multiple findings of local adaptation among marine species with a high gene flow support the possibility of local adaptation among Alaskan red king crab populations (Clucas et al. 2019; Lovrich 2014; Tigano and Friesen 2016; Wilder et al. 2020). Local adaptation could be particularly strong in red king crabs at their postlarval settlement stage where mortality is especially high (Stevens 2014a).

Future sampling strategies must be purposefully developed and carefully planned. For example, we strongly recommend future studies prioritize collecting phenotypic data—even easily measured phenotypes, such as carapace width, maturity status, mass, parasite load, or substrate associations—in conjunction with genetic sampling to increase power to infer adaptive variation. In addition, our samples were collected on surveys or from commercial catches, both of which capture majority male individuals, whereas sequencing more females would allow for comparisons of population structure between sexes and could reveal patterns such as asymmetric sex‐mediated gene flow. Furthermore, the Bering Sea is a particular focus of Alaskan management agencies, and a follow‐up study focused on sequencing individuals throughout the Bering Sea and increasing sample sizes would better define population structure and demographic relationships in this region. Finally, in contrast with previous genetic surveys (Vulstek et al. 2013), our study failed to find population structure within SEAK, perhaps because the lcWGS method we used relies on relatively large sample sizes (*N* ≥ 15) that we could not obtain for every subpopulation in the region. Future studies should focus on increasing sample sizes in this region.

The substantial population structure we documented generally supports current spatial fisheries management strategies for Alaskan red king crab. The distinct genetic groups we identified are already managed separately by state and federal organizations. For example, the Pribilof Islands and Bristol Bay populations are assessed and managed as separate stocks, despite a previous absence of genetic evidence, and our study now lends genetic support for maintaining these current stock delineations. Further splitting the Norton Sound and Chukchi Sea populations into separately managed stocks may be of benefit, but additional research is necessary to further resolve the potential genetic structure that we observed in this region.

Red king crab conservation and rehabilitation efforts in the past have not only focused on fishery closures but also development and potential implementation of artificial propagation and release strategies (Daly, Swingle, and Eckert 2009). Currently, interest is growing at the federal level and among fishery rights‐holders in using stock enhancement in the Gulf of Alaska as a means to increase abundance after 40 years of no recovery. Unfortunately, recent efforts by NOAA to collect local broodstock in the Gulf of Alaska have been largely unsuccessful (Chris Long, Pers. comm.). Nevertheless, in the interest of taking a responsible approach to stock enhancement (Lorenzen, Leber, and Blankenship 2010) and in concordance with Alaska's Genetic Policy and Invertebrate Genetic Guidelines for Mariculture (Gruenthal, Habich, and Gilk‐Baumer 2024), we strongly suggest first increasing efforts to collect broodstock in the Gulf of Alaska. Given the likelihood of adaptive genetic differences between the Gulf of Alaska and Bering Sea, and membership in different larval drift zones (Alaska Administrative Code, § 5 AAC 41.295 (f) 2001) only after significant attempts to collect broodstock across the Gulf of Alaska have been exhausted should the next closest viable stock—EBS, which is the next most genetically similar population—be considered as a potential source as broodstock for GOA stock enhancement.

## Conflicts of Interest

The authors declare no conflicts of interest.

## Supporting information


Appendix S1

## Data Availability

The data that support the findings of this study are openly available in the National Center for Biotechnology Information Sequence Read Archive at https://www.ncbi.nlm.nih.gov/bioproject/PRJNA1181860, accession **PRJNA1181860**, ID 1181860.
