# Supplementary material for: Whole Genome Sequencing Reveals Substantial Genetic Structure and Evidence of Local Adaptation in Alaskan Red King Crab
Source: Evol Appl. 2024 Dec 31;18(1):e70049. doi: 10.1111/eva.70049 (PMC11686092; doi:10.1111/eva.70049)
Supplement: Supplementary file 1 — Appendix S1 [file EVA-18-e70049-s001.docx]

**Supplementary figures**


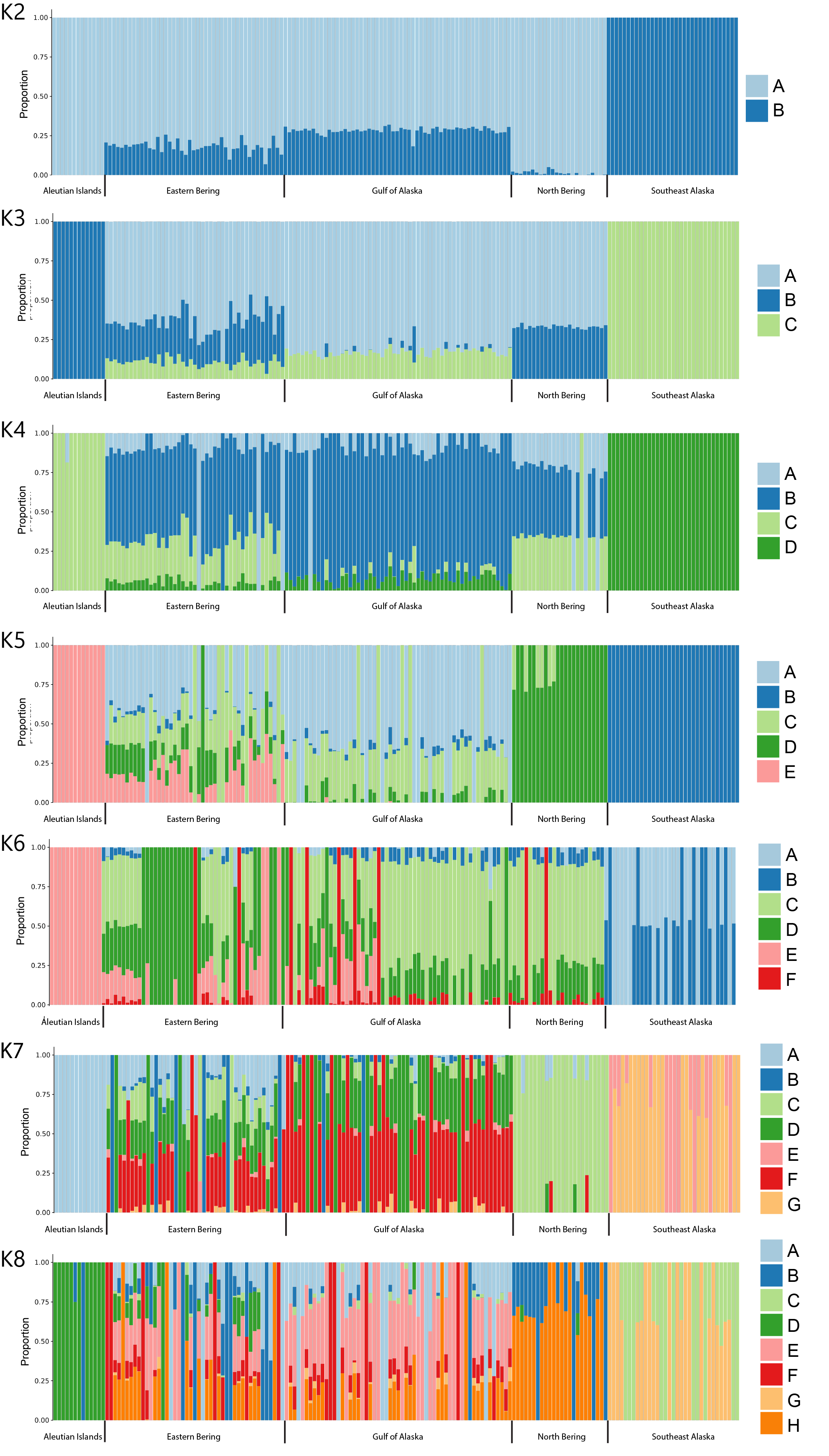


**Figure S1.** Admixture analysis. Admixture was run with K populations = 2 (top) through K = 8 (bottom).

**
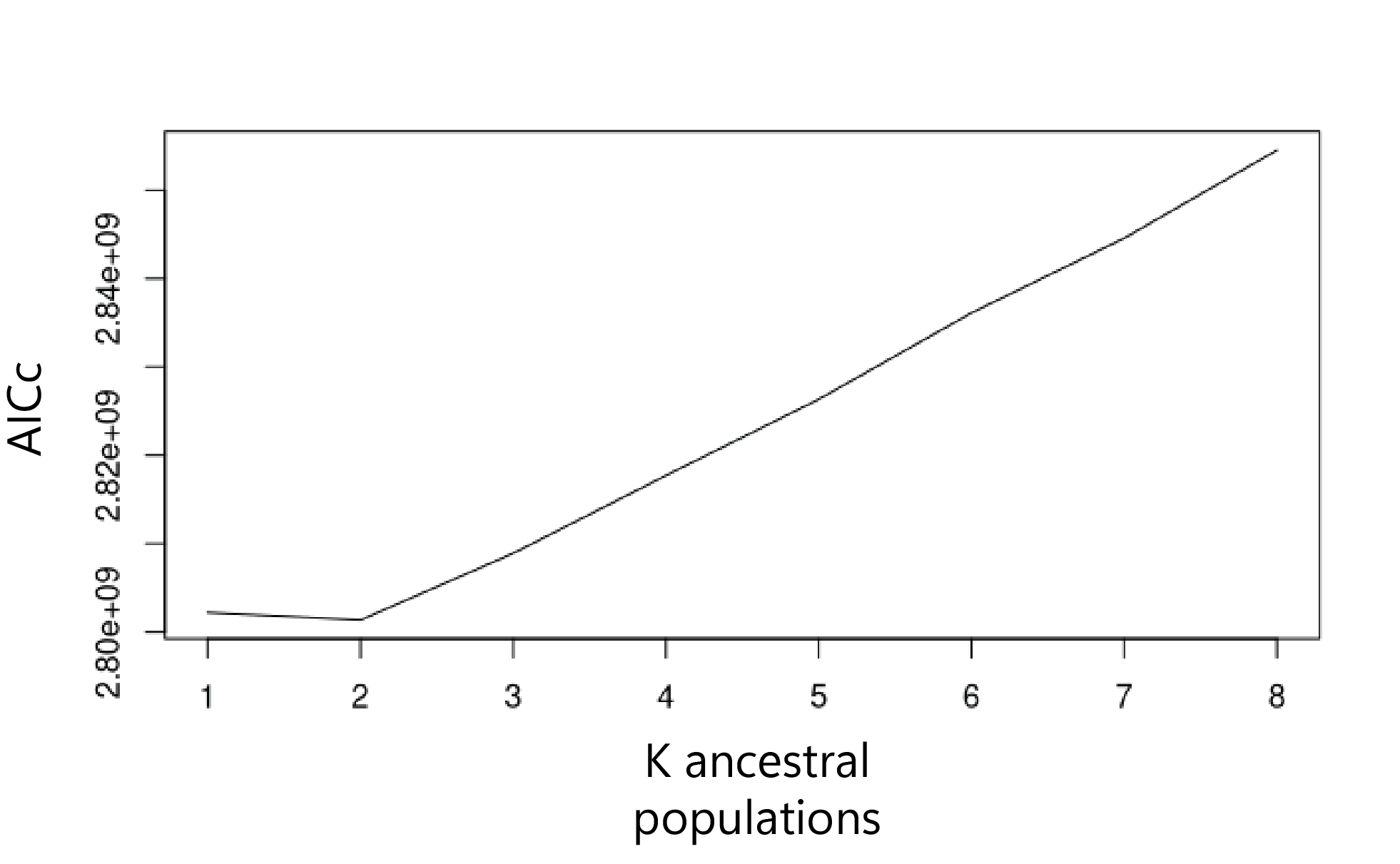
**

**Figure S2.** AICc values plotted on the y-axis against different K ancestral populations of each model tested on the x-axis.

**
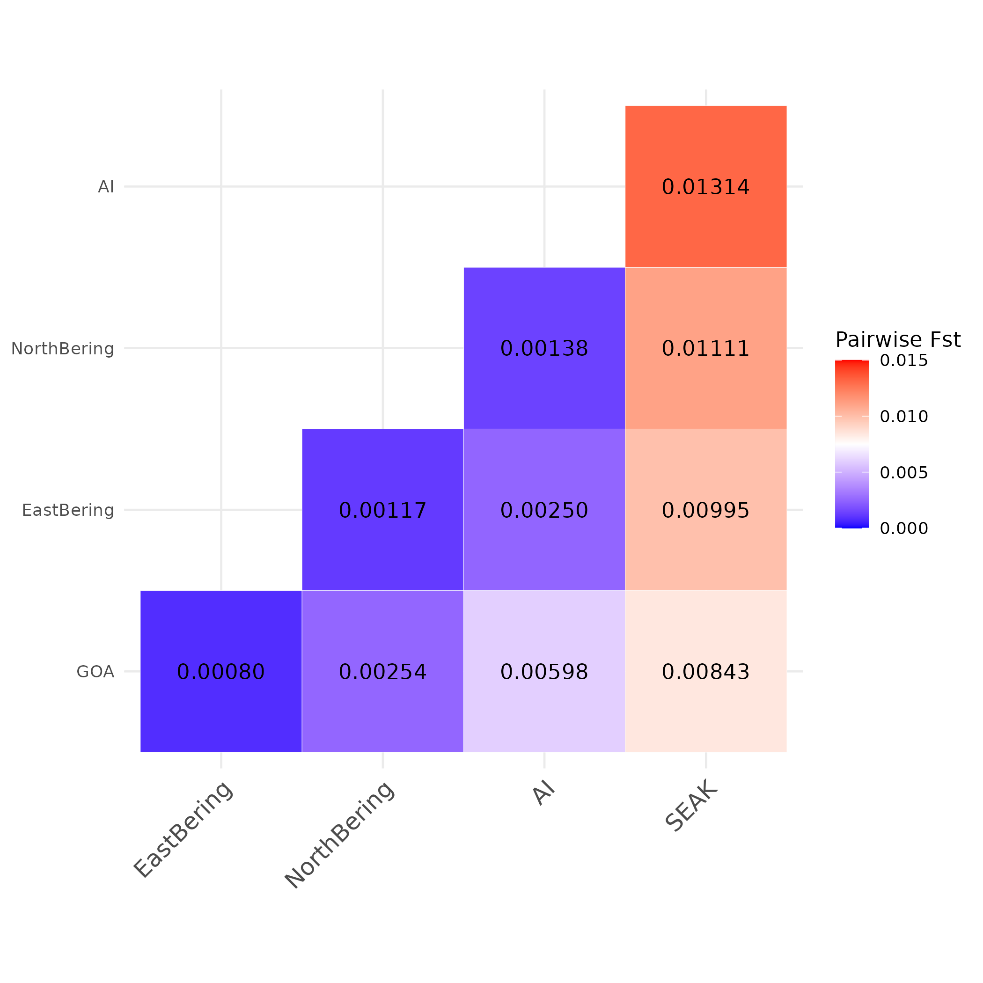
**

**Figure S3.** Pairwise F_ST_ among all populations. Higher F_ST_ values are colored red and lower F_ST_ values are colored blue.

**
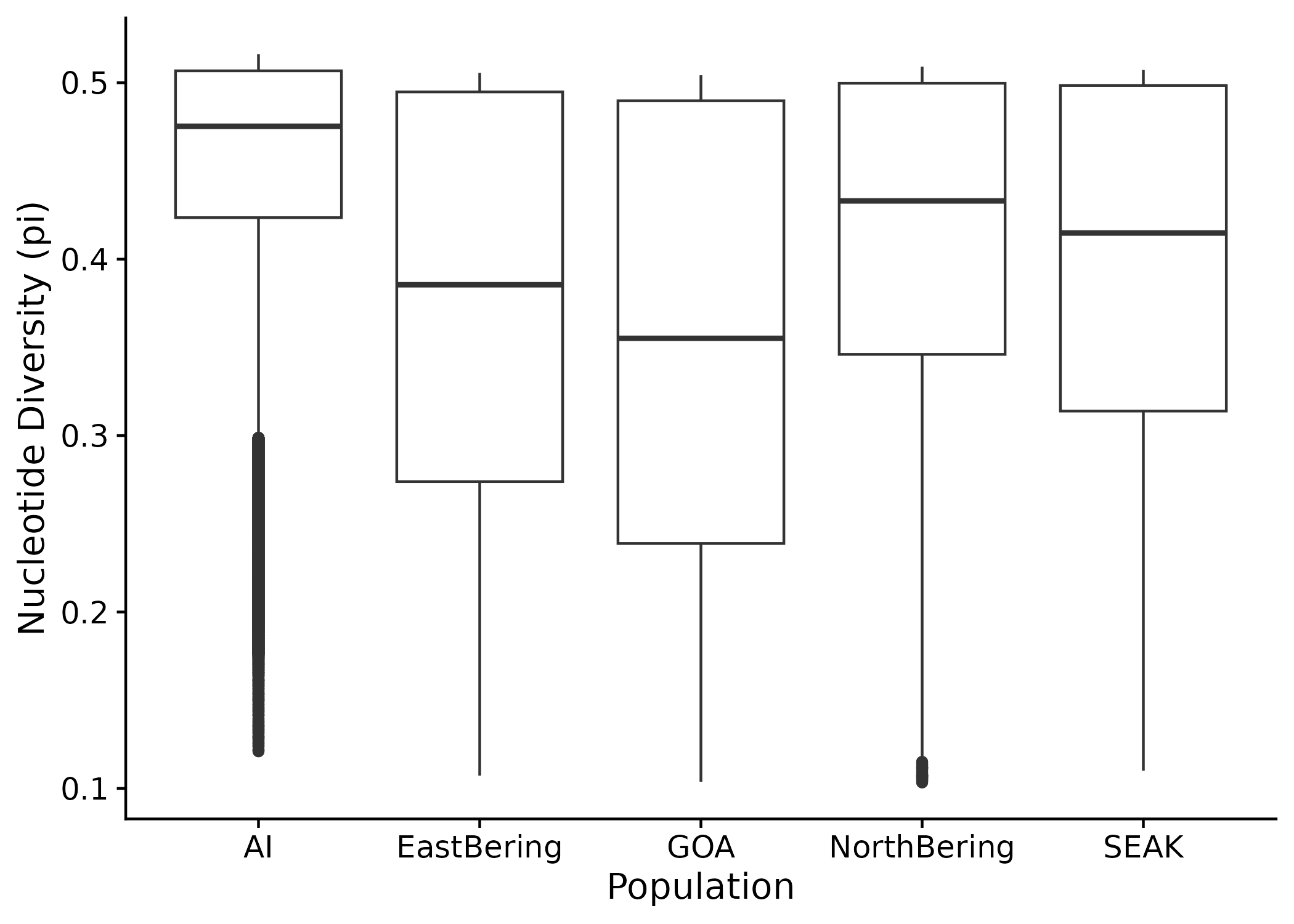
**

**Figure S4.** Global nucleotide diversity by geographic region. AI = Aleutian Islands, EastBering = East Bering Sea, GOA = Gulf of Alaska, NorthBering = North Bering Sea, and SEAK = Southeast Alaska

**
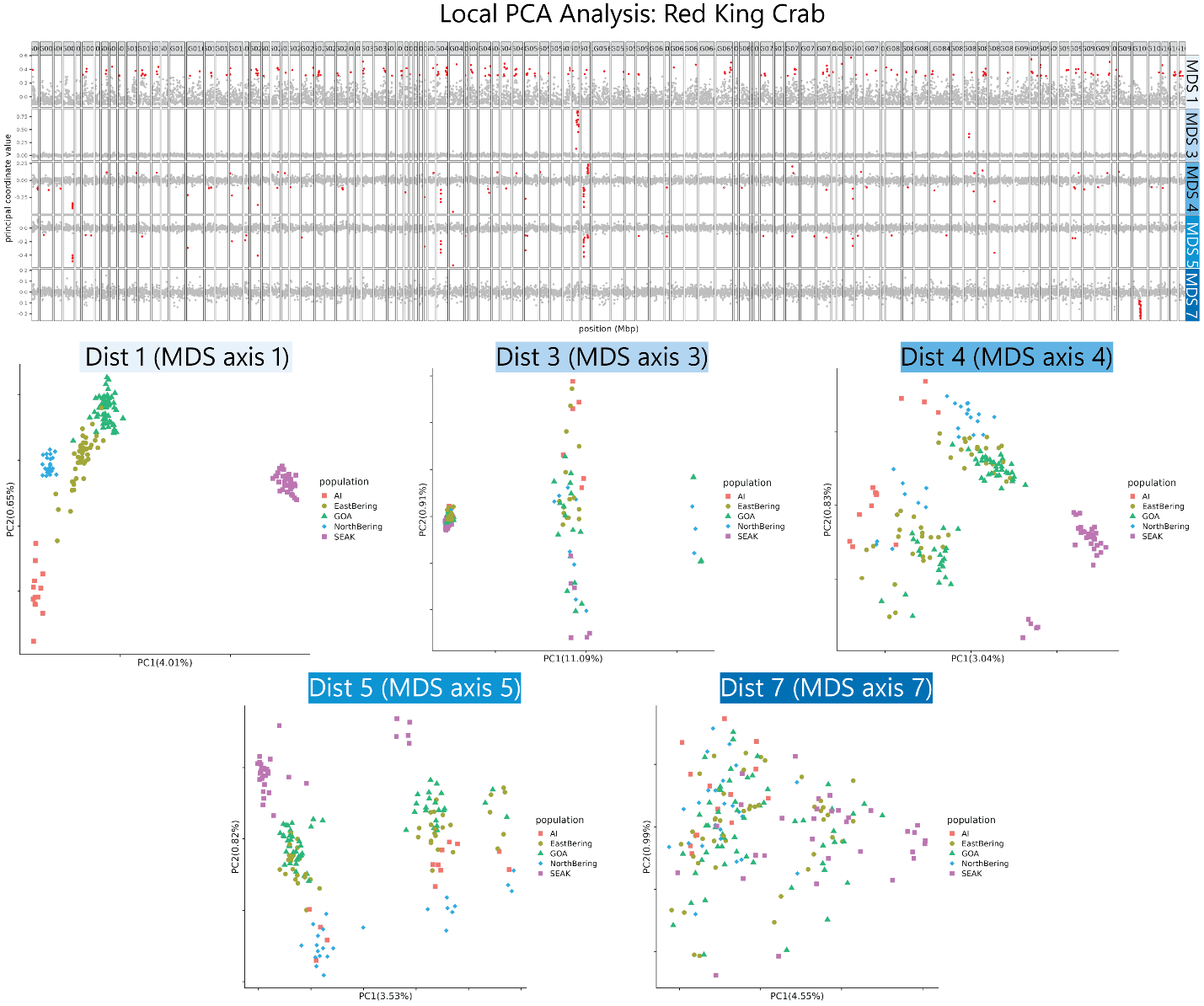
**

**Figure S5.** Local PCA analysis. A) genome scans showing outliers that explain the most variance in the genomic data ordered by MDS axis from top to bottom. The y-axis indicates principal coordinate value of the focal axis. The x-axis is the genomic position. B) PCAs of the outlier regions of each MDS axis. Title colors correspond with the MDS axis in panel A. Each point in the PCA plots represents a single individual. Points are colored by geographic region.

**Supplementary tables**

**Table S1.** Depth filtering parameters for each population.

| Population | Minimum depth filter | Maximum depth filter |
| --- | --- | --- |
| Aleutian Islands | 16 | 320 |
| East Bering | 45 | 900 |
| Gulf of Alaska | 59 | 1180 |
| North Bering | 28 | 560 |
| Southeast Alaska | 35 | 700 |

**Table S2.** List of outlier regions identified by our F_ST_ outlier detection method.

| Chromosome | begin | end | local score peak | population-pair |
| --- | --- | --- | --- | --- |
| 4 | 48525201 | 48536115 | 52.89966 | NBS-AI |
| 4 | 46191145 | 46200755 | 42.23184 | NBS-AI |
| 12 | 84917 | 301294 | 440.0495 | NBS-AI |
| 17 | 18521366 | 18613329 | 22.2787 | NBS-AI |
| 25 | 33167075 | 33168034 | 27.1924 | NBS-AI |
| 100 | 10747869 | 10894263 | 20.69453 | NBS-AI |
| 4 | 48533538 | 48536271 | 65.82577 | NBS-EBS |
| 16 | 74614827 | 74626013 | 85.82441 | NBS-EBS |
| 23 | 30087231 | 30205509 | 27.43969 | NBS-EBS |
| 29 | 35312922 | 35313745 | 31.66798 | NBS-EBS |
| 55 | 30983701 | 30988142 | 63.08854 | NBS-EBS |
| 70 | 40485236 | 40485884 | 50.3087 | NBS-EBS |
| 79 | 66760020 | 66760621 | 27.64148 | NBS-EBS |
| 83 | 24967946 | 24968684 | 23.99325 | NBS-EBS |
| 95 | 25643154 | 25644631 | 42.47903 | NBS-EBS |
| 2 | 37773764 | 37821029 | 38.13646 | NBS-GOA |
| 4 | 46178609 | 46271579 | 76.32969 | NBS-GOA |
| 4 | 48534078 | 48537672 | 46.49216 | NBS-GOA |
| 16 | 74577447 | 74620972 | 99.25196 | NBS-GOA |
| 22 | 14174856 | 14176438 | 46.71674 | NBS-GOA |
| 28 | 40417276 | 40461473 | 51.06677 | NBS-GOA |
| 39 | 13132341 | 13169998 | 165.106 | NBS-GOA |
| 48 | 18114138 | 18124144 | 35.36326 | NBS-GOA |
| 55 | 30975080 | 31021667 | 103.5245 | NBS-GOA |
| 55 | 34853831 | 34968100 | 64.78141 | NBS-GOA |
| 55 | 31085776 | 31125086 | 62.70807 | NBS-GOA |
| 57 | 35729671 | 35774232 | 40.75992 | NBS-GOA |
| 59 | 42654821 | 42730020 | 101.2687 | NBS-GOA |
| 70 | 40485236 | 40485884 | 60.19824 | NBS-GOA |
| 95 | 25643083 | 25656561 | 48.13043 | NBS-GOA |
| 100 | 10747909 | 10894263 | 76.20872 | NBS-GOA |
| 73 | 3022223 | 3340991 | 110.2354 | NBS-SEAK |
| 77 | 32216254 | 32660161 | 166.2596 | NBS-SEAK |
| 81 | 24381572 | 24427857 | 92.7745 | NBS-SEAK |
| 92 | 16362783 | 16467435 | 81.41048 | NBS-SEAK |
| 96 | 38103246 | 38181203 | 161.53 | NBS-SEAK |
| 12 | 154346 | 162886 | 41.11707 | AI-EBS |
| 12 | 207724 | 210422 | 39.80201 | AI-EBS |
| 19 | 34689987 | 34700700 | 36.11057 | AI-EBS |
| 35 | 10870325 | 10881415 | 33.05222 | AI-EBS |
| Chromosome | begin | end | local score peak | population-pair |
| 46 | 54825545 | 54831449 | 29.52229 | AI-EBS |
| 53 | 21050149 | 21050613 | 30.84594 | AI-EBS |
| 59 | 51643656 | 51667367 | 41.50385 | AI-EBS |
| 67 | 10807930 | 10815292 | 40.46006 | AI-EBS |
| 101 | 24144107 | 24253532 | 68.4351 | AI-EBS |
| 1 | 13732794 | 13768856 | 81.86005 | AI-GOA |
| 12 | 154346 | 301471 | 248.172 | AI-GOA |
| 35 | 10870325 | 10881421 | 87.53138 | AI-GOA |
| 81 | 13919860 | 14087111 | 93.68546 | AI-GOA |
| 101 | 24108808 | 24255708 | 152.8528 | AI-GOA |
| 12 | 84917 | 301471 | 616.6686 | AI-SEAK |
| 27 | 7331207 | 7509050 | 99.56822 | AI-SEAK |
| 29 | 28800012 | 29291228 | 207.5919 | AI-SEAK |
| 39 | 17856751 | 17875195 | 134.0527 | AI-SEAK |
| 82 | 11581193 | 11594028 | 77.17805 | AI-SEAK |
| 101 | 24108970 | 24255708 | 150.15 | AI-SEAK |
| 1 | 27258467 | 27335491 | 24.9863 | EBS-GOA |
| 24 | 14889458 | 14912290 | 25.75752 | EBS-GOA |
| 88 | 31978511 | 31980269 | 25.0249 | EBS-GOA |
| 100 | 10750169 | 10858955 | 34.06087 | EBS-GOA |
| 12 | 152445 | 239142 | 148.5937 | EBS-SEAK |
| 25 | 15253426 | 15697811 | 148.4995 | EBS-SEAK |
| 39 | 17868720 | 17879081 | 118.7299 | EBS-SEAK |
| 51 | 12708487 | 12893151 | 137.1272 | EBS-SEAK |
| 56 | 8918518 | 9378709 | 171.0172 | EBS-SEAK |
| 73 | 3022223 | 3340991 | 123.8837 | EBS-SEAK |
| 77 | 32216141 | 32732343 | 301.5612 | EBS-SEAK |
| 82 | 13348857 | 13546166 | 111.6197 | EBS-SEAK |
| 96 | 38103246 | 38181384 | 450.3112 | EBS-SEAK |
| 51 | 12708487 | 12893151 | 133.2533 | GOA-SEAK |
| 75 | 6843901 | 6860148 | 109.9783 | GOA-SEAK |
| 77 | 32216141 | 32718881 | 202.4286 | GOA-SEAK |
| 82 | 13348463 | 13546166 | 137.955 | GOA-SEAK |
| 96 | 38103246 | 38181384 | 363.6971 | GOA-SEAK |
| 104 | 13620704 | 13629318 | 162.0058 | GOA-SEAK |

**Table S3.** Nucleotide-nucleotide blast hits for SR49

| Subject ID | Perc Identity | length | mismatches | gap opens | query start | query end | subject start | subject end | E value | Max Score | description | |
| --- | --- | --- | --- | --- | --- | --- | --- | --- | --- | --- | --- | --- |
| AC140788.4 | 71.712 | 403 | 86 | 12 | 10749344 | 10749729 | 41781 | 41390 | 1.03E-40 | 185 | [Mus musculus BAC clone RP23-452K24 from chromosome 2, complete sequence](https://www.ncbi.nlm.nih.gov/nuccore/AC140788.4) |  |
| AL929262.22 | 71.712 | 403 | 86 | 12 | 10749344 | 10749729 | 6015 | 5624 | 1.03E-40 | 185 | [Mouse DNA sequence from clone RP23-452K24 on chromosome 2, complete sequence](https://www.ncbi.nlm.nih.gov/nuccore/AL929262.22) |  |
| OW971800.1 | 78.788 | 66 | 14 | 0 | 10749669 | 10749734 | 37128695 | 37128760 | 0.036 | 57.2 | [Mus musculus genome assembly, chromosome: 17](https://www.ncbi.nlm.nih.gov/nuccore/OW971800.1) |  |
| AC122005.3 | 67.532 | 693 | 131 | 37 | 10816753 | 10817413 | 143984 | 144614 | 1.74E-31 | 154 | [Mus musculus chromosome 5 clone RP24-340B11, complete sequence](https://www.ncbi.nlm.nih.gov/nuccore/AC122005.3) |  |
| AC124392.6 | 71.875 | 640 | 123 | 28 | 10816835 | 10817464 | 47814 | 48406 | 1.10E-65 | 269 | [Mus musculus BAC clone RP24-441F24 from chromosome 13, complete sequence](https://www.ncbi.nlm.nih.gov/nuccore/AC124392.6) |  |
| BX510300.12 | 69.871 | 541 | 112 | 28 | 10816925 | 10817456 | 104017 | 103519 | 1.17E-33 | 161 | [Mouse DNA sequence from clone RP23-221A16 on chromosome 4, complete sequence](https://www.ncbi.nlm.nih.gov/nuccore/BX510300.12) |  |
| AC102039.8 | 69.414 | 546 | 95 | 25 | 10816970 | 10817495 | 121710 | 121217 | 1.03E-40 | 185 | [Mus musculus chromosome 5, clone RP23-262L7, complete sequence](https://www.ncbi.nlm.nih.gov/nuccore/AC102039.8) |  |
| AC102056.17 | 69.797 | 543 | 98 | 26 | 10816970 | 10817495 | 170355 | 170848 | 3.59E-40 | 183 | [Mus musculus chromosome 5, clone RP23-190H2, complete sequence](https://www.ncbi.nlm.nih.gov/nuccore/AC102056.17) |  |
| AC102039.8 | 69.209 | 354 | 71 | 15 | 10817130 | 10817467 | 121694 | 121363 | 1.53E-19 | 114 | [Mus musculus chromosome 5, clone RP23-262L7, complete sequence](https://www.ncbi.nlm.nih.gov/nuccore/AC102039.8) |  |
| OX389811.1 | 73.203 | 306 | 69 | 9 | 10817168 | 10817466 | 56257041 | 56256742 | 1.74E-31 | 154 | [Mus musculus genome assembly, chromosome: 17](https://www.ncbi.nlm.nih.gov/nuccore/OX389811.1) |  |
| OW971800.1 | 73.203 | 306 | 69 | 9 | 10817168 | 10817466 | 50127789 | 50127490 | 1.74E-31 | 154 | [Mouse DNA sequence from clone RP23-452K24 on chromosome 2, complete sequence](https://www.ncbi.nlm.nih.gov/nuccore/AL929262.22) |  |
| BX510300.12 | 70.637 | 361 | 57 | 23 | 10817171 | 10817501 | 104021 | 103680 | 1.53E-19 | 114 | [Mouse DNA sequence from clone RP23-221A16 on chromosome 4, complete sequence](https://www.ncbi.nlm.nih.gov/nuccore/BX510300.12) |  |
| OX389811.1 | 71.825 | 252 | 45 | 12 | 10817218 | 10817466 | 56257042 | 56256814 | 2.76E-16 | 104 | [Mus musculus genome assembly, chromosome: 17](https://www.ncbi.nlm.nih.gov/nuccore/OX389811.1) |  |
| OW971800.1 | 71.825 | 252 | 45 | 12 | 10817218 | 10817466 | 50127790 | 50127562 | 2.76E-16 | 104 | [Mouse DNA sequence from clone RP23-452K24 on chromosome 2, complete sequence](https://www.ncbi.nlm.nih.gov/nuccore/AL929262.22) |  |
| AC124392.6 | 69.951 | 203 | 45 | 7 | 10817268 | 10817467 | 48574 | 48763 | 1.34E-07 | 75.2 | [Mus musculus BAC clone RP24-441F24 from chromosome 13, complete sequence](https://www.ncbi.nlm.nih.gov/nuccore/AC124392.6) |  |
| OX389811.1 | 72.34 | 188 | 38 | 10 | 10817315 | 10817495 | 56257059 | 56256879 | 4.68E-07 | 73.4 | [Mus musculus genome assembly, chromosome: 17](https://www.ncbi.nlm.nih.gov/nuccore/OX389811.1) |  |
| OW971800.1 | 72.34 | 188 | 38 | 10 | 10817315 | 10817495 | 50127807 | 50127627 | 4.68E-07 | 73.4 | [Mouse DNA sequence from clone RP23-452K24 on chromosome 2, complete sequence](https://www.ncbi.nlm.nih.gov/nuccore/AL929262.22) |  |
| OX359226.1 | 79.518 | 166 | 33 | 1 | 10840969 | 10841134 | 12315244 | 12315080 | 3.15E-28 | 143 | [Bicyclus anynana genome assembly, chromosome: 22](https://www.ncbi.nlm.nih.gov/nuccore/OX359226.1) |  |
| XM_024098218.2 | 79.87 | 154 | 31 | 0 | 10840981 | 10841134 | 460 | 613 | 1.34E-26 | 139 | PREDICTED: glutamate-gated chloride channel, (LOC112057699), transcript variant X11 |  |
| XM_024098217.2 | 79.87 | 154 | 31 | 0 | 10840981 | 10841134 | 463 | 616 | 1.34E-26 | 139 | PREDICTED: glutamate-gated chloride channel, (LOC112057699), transcript variant X10 |  |
| XM_024098216.2 | 79.87 | 154 | 31 | 0 | 10840981 | 10841134 | 466 | 619 | 1.34E-26 | 139 | PREDICTED: glutamate-gated chloride channel, (LOC112057699), transcript variant X9 |  |
| XM_052888342.1 | 79.87 | 154 | 31 | 0 | 10840981 | 10841134 | 460 | 613 | 1.34E-26 | 139 | PREDICTED: glutamate-gated chloride channel, (LOC112057699), transcript variant X8 |  |
| XM_024098214.2 | 79.87 | 154 | 31 | 0 | 10840981 | 10841134 | 460 | 613 | 1.34E-26 | 139 | PREDICTED: glutamate-gated chloride channel, (LOC112057699), transcript variant X7 |  |
| XM_052888341.1 | 79.87 | 154 | 31 | 0 | 10840981 | 10841134 | 463 | 616 | 1.34E-26 | 139 | PREDICTED: glutamate-gated chloride channel, (LOC112057699), transcript variant X6 |  |
| XM_024098215.2 | 79.87 | 154 | 31 | 0 | 10840981 | 10841134 | 460 | 613 | 1.34E-26 | 139 | PREDICTED: glutamate-gated chloride channel, (LOC112057699), transcript variant X5 |  |
| XM_024098213.2 | 79.87 | 154 | 31 | 0 | 10840981 | 10841134 | 466 | 619 | 1.34E-26 | 139 | PREDICTED: glutamate-gated chloride channel, (LOC112057699), transcript variant X4 |  |
| XM_024098212.2 | 79.87 | 154 | 31 | 0 | 10840981 | 10841134 | 463 | 616 | 1.34E-26 | 139 | PREDICTED: glutamate-gated chloride channel, (LOC112057699), transcript variant X3 |  |
| XM_024098210.2 | 79.87 | 154 | 31 | 0 | 10840981 | 10841134 | 552 | 705 | 1.34E-26 | 139 | PREDICTED: glutamate-gated chloride channel, (LOC112057699), transcript variant X2 |  |
| XM_024098209.2 | 79.87 | 154 | 31 | 0 | 10840981 | 10841134 | 466 | 619 | 1.34E-26 | 139 | PREDICTED: glutamate-gated chloride channel, (LOC112057699), transcript variant X1 |  |
| XM_027351827.1 | 77.124 | 153 | 35 | 0 | 10840981 | 10841133 | 130 | 282 | 1.26E-20 | 119 | PREDICTED: Penaeus vannamei glutamate-gated chloride channel-like (LOC113801023), mRNA |  |
| XM_027377726.1 | 87.333 | 150 | 19 | 0 | 10840988 | 10841137 | 1 | 150 | 1.03E-40 | 186 | PREDICTED: Penaeus vannamei glutamate-gated chloride channel-like |  |
| XM_027377726.1 | 78.049 | 82 | 18 | 0 | 10842316 | 10842397 | 149 | 230 | 1.99E-05 | 68 | PREDICTED: Penaeus vannamei glutamate-gated chloride channel-like |  |
| XM_024098218.2 | 87.931 | 58 | 7 | 0 | 10842318 | 10842375 | 617 | 674 | 4.68E-07 | 74.3 | PREDICTED: glutamate-gated chloride channel, (LOC112057699), transcript variant X11 |  |
| XM_024098217.2 | 87.931 | 58 | 7 | 0 | 10842318 | 10842375 | 620 | 677 | 4.68E-07 | 74.3 | PREDICTED: glutamate-gated chloride channel, (LOC112057699), transcript variant X10 |  |
| XM_024098216.2 | 87.931 | 58 | 7 | 0 | 10842318 | 10842375 | 623 | 680 | 4.68E-07 | 74.3 | PREDICTED: glutamate-gated chloride channel, (LOC112057699), transcript variant X9 |  |
| XM_052888342.1 | 87.931 | 58 | 7 | 0 | 10842318 | 10842375 | 617 | 674 | 4.68E-07 | 74.3 | PREDICTED: glutamate-gated chloride channel, (LOC112057699), transcript variant X8 |  |
| XM_024098214.2 | 87.931 | 58 | 7 | 0 | 10842318 | 10842375 | 617 | 674 | 4.68E-07 | 74.3 | PREDICTED: glutamate-gated chloride channel, (LOC112057699), transcript variant X7 |  |
| XM_052888341.1 | 87.931 | 58 | 7 | 0 | 10842318 | 10842375 | 620 | 677 | 4.68E-07 | 74.3 | PREDICTED: glutamate-gated chloride channel, (LOC112057699), transcript variant X6 |  |
| XM_024098215.2 | 87.931 | 58 | 7 | 0 | 10842318 | 10842375 | 617 | 674 | 4.68E-07 | 74.3 | PREDICTED: glutamate-gated chloride channel, (LOC112057699), transcript variant X5 |  |
| XM_024098213.2 | 87.931 | 58 | 7 | 0 | 10842318 | 10842375 | 623 | 680 | 4.68E-07 | 74.3 | PREDICTED: glutamate-gated chloride channel, (LOC112057699), transcript variant X4 |  |
| XM_024098212.2 | 87.931 | 58 | 7 | 0 | 10842318 | 10842375 | 620 | 677 | 4.68E-07 | 74.3 | PREDICTED: glutamate-gated chloride channel, (LOC112057699), transcript variant X3 |  |
| XM_024098210.2 | 87.931 | 58 | 7 | 0 | 10842318 | 10842375 | 709 | 766 | 4.68E-07 | 74.3 | PREDICTED: glutamate-gated chloride channel, (LOC112057699), transcript variant X2 |  |
| XM_024098209.2 | 87.931 | 58 | 7 | 0 | 10842318 | 10842375 | 623 | 680 | 4.68E-07 | 74.3 | PREDICTED: glutamate-gated chloride channel, (LOC112057699), transcript variant X1 |  |
| OX359226.1 | 78.022 | 91 | 20 | 0 | 10842318 | 10842408 | 12315076 | 12314986 | 1.34E-07 | 75.2 | Bicyclus anynana genome assembly, chromosome: 22 |  |
| OX359226.1 | 77.67 | 103 | 23 | 0 | 10843581 | 10843683 | 12312511 | 12312409 | 9.04E-10 | 83.3 | Bicyclus anynana genome assembly, chromosome: 22 |  |
| XM_024098218.2 | 77 | 100 | 23 | 0 | 10843584 | 10843683 | 699 | 798 | 3.84E-08 | 77.9 | PREDICTED: glutamate-gated chloride channel, (LOC112057699), transcript variant X11 |  |
| XM_024098217.2 | 77 | 100 | 23 | 0 | 10843584 | 10843683 | 702 | 801 | 3.84E-08 | 77.9 | PREDICTED: glutamate-gated chloride channel, (LOC112057699), transcript variant X10 |  |
| XM_024098216.2 | 77 | 100 | 23 | 0 | 10843584 | 10843683 | 705 | 804 | 3.84E-08 | 77.9 | PREDICTED: glutamate-gated chloride channel, (LOC112057699), transcript variant X9 |  |
| XM_052888342.1 | 77 | 100 | 23 | 0 | 10843584 | 10843683 | 699 | 798 | 3.84E-08 | 77.9 | PREDICTED: glutamate-gated chloride channel, (LOC112057699), transcript variant X8 |  |
| XM_024098214.2 | 77 | 100 | 23 | 0 | 10843584 | 10843683 | 699 | 798 | 3.84E-08 | 77.9 | PREDICTED: glutamate-gated chloride channel, (LOC112057699), transcript variant X7 |  |
| XM_052888341.1 | 77 | 100 | 23 | 0 | 10843584 | 10843683 | 702 | 801 | 3.84E-08 | 77.9 | PREDICTED: glutamate-gated chloride channel, (LOC112057699), transcript variant X6 |  |
| XM_024098215.2 | 77 | 100 | 23 | 0 | 10843584 | 10843683 | 699 | 798 | 3.84E-08 | 77.9 | PREDICTED: glutamate-gated chloride channel, (LOC112057699), transcript variant X5 |  |
| XM_024098213.2 | 77 | 100 | 23 | 0 | 10843584 | 10843683 | 705 | 804 | 3.84E-08 | 77.9 | PREDICTED: glutamate-gated chloride channel, (LOC112057699), transcript variant X4 |  |
| XM_024098212.2 | 77 | 100 | 23 | 0 | 10843584 | 10843683 | 702 | 801 | 3.84E-08 | 77.9 | PREDICTED: glutamate-gated chloride channel, (LOC112057699), transcript variant X3 |  |
| XM_024098210.2 | 77 | 100 | 23 | 0 | 10843584 | 10843683 | 791 | 890 | 3.84E-08 | 77.9 | PREDICTED: glutamate-gated chloride channel, (LOC112057699), transcript variant X2 |  |
| XM_024098209.2 | 77 | 100 | 23 | 0 | 10843584 | 10843683 | 705 | 804 | 3.84E-08 | 77.9 | PREDICTED: glutamate-gated chloride channel, (LOC112057699), transcript variant X1 |  |
| XM_027377726.1 | 82.796 | 93 | 16 | 0 | 10843586 | 10843678 | 235 | 327 | 4.10E-14 | 96.9 | PREDICTED: Penaeus vannamei glutamate-gated chloride channel-like |  |
| XM_027367309.1 | 77.551 | 98 | 22 | 0 | 10843586 | 10843683 | 25 | 122 | 1.10E-08 | 78.8 | PREDICTED: Penaeus vannamei glutamate-gated chloride channel-like |  |
| XM_027351827.1 | 72.059 | 136 | 38 | 0 | 10843586 | 10843721 | 371 | 506 | 1.34E-07 | 75.2 | PREDICTED: Penaeus vannamei glutamate-gated chloride channel-like (LOC113801023), mRNA |  |
| XM_024098218.2 | 77.778 | 225 | 50 | 0 | 10852262 | 10852486 | 838 | 1062 | 1.25E-39 | 181 | PREDICTED: glutamate-gated chloride channel, (LOC112057699), transcript variant X11 |  |
| XM_024098217.2 | 77.778 | 225 | 50 | 0 | 10852262 | 10852486 | 841 | 1065 | 1.25E-39 | 181 | PREDICTED: glutamate-gated chloride channel, (LOC112057699), transcript variant X10 |  |
| XM_024098216.2 | 77.778 | 225 | 50 | 0 | 10852262 | 10852486 | 844 | 1068 | 1.25E-39 | 181 | PREDICTED: glutamate-gated chloride channel, (LOC112057699), transcript variant X9 |  |
| XM_052888342.1 | 77.778 | 225 | 50 | 0 | 10852262 | 10852486 | 838 | 1062 | 1.25E-39 | 181 | PREDICTED: glutamate-gated chloride channel, (LOC112057699), transcript variant X8 |  |
| XM_024098214.2 | 77.778 | 225 | 50 | 0 | 10852262 | 10852486 | 838 | 1062 | 1.25E-39 | 181 | PREDICTED: glutamate-gated chloride channel, (LOC112057699), transcript variant X7 |  |
| XM_052888341.1 | 77.778 | 225 | 50 | 0 | 10852262 | 10852486 | 841 | 1065 | 1.25E-39 | 181 | PREDICTED: glutamate-gated chloride channel, (LOC112057699), transcript variant X6 |  |
| XM_024098215.2 | 77.778 | 225 | 50 | 0 | 10852262 | 10852486 | 838 | 1062 | 1.25E-39 | 181 | PREDICTED: glutamate-gated chloride channel, (LOC112057699), transcript variant X5 |  |
| XM_024098213.2 | 77.778 | 225 | 50 | 0 | 10852262 | 10852486 | 844 | 1068 | 1.25E-39 | 181 | PREDICTED: glutamate-gated chloride channel, (LOC112057699), transcript variant X4 |  |
| XM_024098212.2 | 77.778 | 225 | 50 | 0 | 10852262 | 10852486 | 841 | 1065 | 1.25E-39 | 181 | PREDICTED: glutamate-gated chloride channel, (LOC112057699), transcript variant X3 |  |
| XM_024098210.2 | 77.778 | 225 | 50 | 0 | 10852262 | 10852486 | 930 | 1154 | 1.25E-39 | 181 | PREDICTED: glutamate-gated chloride channel, (LOC112057699), transcript variant X2 |  |
| XM_024098209.2 | 77.778 | 225 | 50 | 0 | 10852262 | 10852486 | 844 | 1068 | 1.25E-39 | 181 | PREDICTED: glutamate-gated chloride channel, (LOC112057699), transcript variant X1 |  |
| XM_021848587.1 | 76.522 | 230 | 54 | 0 | 10852262 | 10852491 | 1360 | 1589 | 6.50E-37 | 172 | PREDICTED: Aedes aegypti glutamate-gated chloride channel (LOC5580270), transcript variant X16 |  |
| XM_021848586.1 | 76.522 | 230 | 54 | 0 | 10852262 | 10852491 | 707 | 936 | 6.50E-37 | 172 | PREDICTED: Aedes aegypti glutamate-gated chloride channel (LOC5580270), transcript variant X15 |  |
| XM_021848585.1 | 76.522 | 230 | 54 | 0 | 10852262 | 10852491 | 1360 | 1589 | 6.50E-37 | 172 | PREDICTED: Aedes aegypti glutamate-gated chloride channel (LOC5580270), transcript variant X14 |  |
| XM_021848583.1 | 76.522 | 230 | 54 | 0 | 10852262 | 10852491 | 1363 | 1592 | 6.50E-37 | 172 | PREDICTED: Aedes aegypti glutamate-gated chloride channel (LOC5580270), transcript variant X13 |  |
| XM_021848582.1 | 76.522 | 230 | 54 | 0 | 10852262 | 10852491 | 1364 | 1593 | 6.50E-37 | 172 | PREDICTED: Aedes aegypti glutamate-gated chloride channel (LOC5580270), transcript variant X12 |  |
| XM_021848581.1 | 76.522 | 230 | 54 | 0 | 10852262 | 10852491 | 1360 | 1589 | 6.50E-37 | 172 | PREDICTED: Aedes aegypti glutamate-gated chloride channel (LOC5580270), transcript variant X11 |  |
| XM_001662847.2 | 76.522 | 230 | 54 | 0 | 10852262 | 10852491 | 1360 | 1589 | 6.50E-37 | 172 | PREDICTED: Aedes aegypti glutamate-gated chloride channel (LOC5580270), transcript variant X10 |  |
| XM_021848580.1 | 76.522 | 230 | 54 | 0 | 10852262 | 10852491 | 1363 | 1592 | 6.50E-37 | 172 | PREDICTED: Aedes aegypti glutamate-gated chloride channel (LOC5580270), transcript variant X9 |  |
| XM_021848579.1 | 76.522 | 230 | 54 | 0 | 10852262 | 10852491 | 1365 | 1594 | 6.50E-37 | 172 | PREDICTED: Aedes aegypti glutamate-gated chloride channel (LOC5580270), transcript variant X8 |  |
| XM_021848578.1 | 76.522 | 230 | 54 | 0 | 10852262 | 10852491 | 1360 | 1589 | 6.50E-37 | 172 | PREDICTED: Aedes aegypti glutamate-gated chloride channel (LOC5580270), transcript variant X7 |  |
| XM_021848577.1 | 76.522 | 230 | 54 | 0 | 10852262 | 10852491 | 1360 | 1589 | 6.50E-37 | 172 | PREDICTED: Aedes aegypti glutamate-gated chloride channel (LOC5580270), transcript variant X6 |  |
| XM_021848576.1 | 76.522 | 230 | 54 | 0 | 10852262 | 10852491 | 1364 | 1593 | 6.50E-37 | 172 | PREDICTED: Aedes aegypti glutamate-gated chloride channel (LOC5580270), transcript variant X5 |  |
| XM_021848575.1 | 76.522 | 230 | 54 | 0 | 10852262 | 10852491 | 1424 | 1653 | 6.50E-37 | 172 | PREDICTED: Aedes aegypti glutamate-gated chloride channel (LOC5580270), transcript variant X4 |  |
| XM_021848574.1 | 76.522 | 230 | 54 | 0 | 10852262 | 10852491 | 1425 | 1654 | 6.50E-37 | 172 | PREDICTED: Aedes aegypti glutamate-gated chloride channel (LOC5580270), transcript variant X3 |  |
| XM_021848573.1 | 76.522 | 230 | 54 | 0 | 10852262 | 10852491 | 1426 | 1655 | 6.50E-37 | 172 | PREDICTED: Aedes aegypti glutamate-gated chloride channel (LOC5580270), transcript variant X2 |  |
| XM_021848572.1 | 76.522 | 230 | 54 | 0 | 10852262 | 10852491 | 1428 | 1657 | 6.50E-37 | 172 | PREDICTED: Aedes aegypti glutamate-gated chloride channel (LOC5580270), transcript variant X1 |  |
| OX359226.1 | 82.667 | 150 | 26 | 0 | 10852262 | 10852411 | 12309218 | 12309069 | 1.74E-31 | 154 | Bicyclus anynana genome assembly, chromosome: 22 |  |
| XM_027367309.1 | 78.222 | 225 | 47 | 2 | 10852268 | 10852491 | 168 | 391 | 1.53E-38 | 178 | PREDICTED: Penaeus vannamei glutamate-gated chloride channel-like |  |
| XM_027351827.1 | 76.682 | 223 | 52 | 0 | 10852269 | 10852491 | 515 | 737 | 7.91E-36 | 168 | PREDICTED: Penaeus vannamei glutamate-gated chloride channel-like |  |
| XM_021848587.1 | 81.818 | 88 | 16 | 0 | 10853609 | 10853696 | 1584 | 1671 | 2.13E-11 | 87.8 | PREDICTED: Aedes aegypti glutamate-gated chloride channel (LOC5580270), transcript variant X16, mRNA |  |
| XM_021848586.1 | 81.818 | 88 | 16 | 0 | 10853609 | 10853696 | 931 | 1018 | 2.13E-11 | 87.8 | PREDICTED: Aedes aegypti glutamate-gated chloride channel (LOC5580270), transcript variant X15, mRNA |  |
| XM_021848585.1 | 81.818 | 88 | 16 | 0 | 10853609 | 10853696 | 1584 | 1671 | 2.13E-11 | 87.8 | PREDICTED: Aedes aegypti glutamate-gated chloride channel (LOC5580270), transcript variant X14, mRNA |  |
| XM_021848583.1 | 81.818 | 88 | 16 | 0 | 10853609 | 10853696 | 1587 | 1674 | 2.13E-11 | 87.8 | PREDICTED: Aedes aegypti glutamate-gated chloride channel (LOC5580270), transcript variant X13, mRNA |  |
| XM_021848582.1 | 81.818 | 88 | 16 | 0 | 10853609 | 10853696 | 1588 | 1675 | 2.13E-11 | 87.8 | PREDICTED: Aedes aegypti glutamate-gated chloride channel (LOC5580270), transcript variant X12, mRNA |  |
| XM_021848581.1 | 81.818 | 88 | 16 | 0 | 10853609 | 10853696 | 1584 | 1671 | 2.13E-11 | 87.8 | PREDICTED: Aedes aegypti glutamate-gated chloride channel (LOC5580270), transcript variant X11, mRNA |  |
| XM_001662847.2 | 81.818 | 88 | 16 | 0 | 10853609 | 10853696 | 1584 | 1671 | 2.13E-11 | 87.8 | PREDICTED: Aedes aegypti glutamate-gated chloride channel (LOC5580270), transcript variant X10, mRNA |  |
| XM_021848580.1 | 81.818 | 88 | 16 | 0 | 10853609 | 10853696 | 1587 | 1674 | 2.13E-11 | 87.8 | PREDICTED: Aedes aegypti glutamate-gated chloride channel (LOC5580270), transcript variant X9, mRNA |  |
| XM_021848579.1 | 81.72 | 93 | 17 | 0 | 10853609 | 10853701 | 1589 | 1681 | 1.74E-12 | 92.4 | PREDICTED: Aedes aegypti glutamate-gated chloride channel (LOC5580270), transcript variant X8, mRNA |  |
| XM_021848578.1 | 81.818 | 88 | 16 | 0 | 10853609 | 10853696 | 1584 | 1671 | 2.13E-11 | 87.8 | PREDICTED: Aedes aegypti glutamate-gated chloride channel (LOC5580270), transcript variant X7, mRNA |  |
| XM_021848577.1 | 81.818 | 88 | 16 | 0 | 10853609 | 10853696 | 1584 | 1671 | 2.13E-11 | 87.8 | PREDICTED: Aedes aegypti glutamate-gated chloride channel (LOC5580270), transcript variant X6, mRNA |  |
| XM_021848576.1 | 81.818 | 88 | 16 | 0 | 10853609 | 10853696 | 1588 | 1675 | 2.13E-11 | 87.8 | PREDICTED: Aedes aegypti glutamate-gated chloride channel (LOC5580270), transcript variant X5, mRNA |  |
| XM_021848575.1 | 81.818 | 88 | 16 | 0 | 10853609 | 10853696 | 1648 | 1735 | 2.13E-11 | 87.8 | PREDICTED: Aedes aegypti glutamate-gated chloride channel (LOC5580270), transcript variant X4, mRNA |  |
| XM_021848574.1 | 81.818 | 88 | 16 | 0 | 10853609 | 10853696 | 1649 | 1736 | 2.13E-11 | 87.8 | PREDICTED: Aedes aegypti glutamate-gated chloride channel (LOC5580270), transcript variant X3, mRNA |  |
| XM_021848573.1 | 81.818 | 88 | 16 | 0 | 10853609 | 10853696 | 1650 | 1737 | 2.13E-11 | 87.8 | PREDICTED: Aedes aegypti glutamate-gated chloride channel (LOC5580270), transcript variant X2, mRNA |  |
| XM_021848572.1 | 81.818 | 88 | 16 | 0 | 10853609 | 10853696 | 1652 | 1739 | 2.13E-11 | 87.8 | PREDICTED: Aedes aegypti glutamate-gated chloride channel (LOC5580270), transcript variant X1, mRNA |  |
| XM_027351827.1 | 83.036 | 112 | 19 | 0 | 10853609 | 10853720 | 732 | 843 | 4.38E-20 | 117 | PREDICTED: Penaeus vannamei glutamate-gated chloride channel-like (LOC113801023), mRNA |  |
| XM_027377726.1 | 81.579 | 152 | 22 | 2 | 10853611 | 10853762 | 481 | 626 | 9.03E-29 | 145 | PREDICTED: Penaeus vannamei glutamate-gated chloride channel-like |  |
| XM_024098218.2 | 80.18 | 111 | 22 | 0 | 10853611 | 10853721 | 1064 | 1174 | 9.65E-16 | 102 | PREDICTED: glutamate-gated chloride channel, (LOC112057699), transcript variant X11 |  |
| XM_024098217.2 | 80.18 | 111 | 22 | 0 | 10853611 | 10853721 | 1067 | 1177 | 9.65E-16 | 102 | PREDICTED: glutamate-gated chloride channel, (LOC112057699), transcript variant X10 |  |
| XM_024098216.2 | 80.18 | 111 | 22 | 0 | 10853611 | 10853721 | 1070 | 1180 | 9.65E-16 | 102 | PREDICTED: glutamate-gated chloride channel, (LOC112057699), transcript variant X9 |  |
| XM_052888342.1 | 80.18 | 111 | 22 | 0 | 10853611 | 10853721 | 1064 | 1174 | 9.65E-16 | 102 | PREDICTED: glutamate-gated chloride channel, (LOC112057699), transcript variant X8 |  |
| XM_024098214.2 | 80.18 | 111 | 22 | 0 | 10853611 | 10853721 | 1064 | 1174 | 9.65E-16 | 102 | PREDICTED: glutamate-gated chloride channel, (LOC112057699), transcript variant X7 |  |
| XM_052888341.1 | 80.18 | 111 | 22 | 0 | 10853611 | 10853721 | 1067 | 1177 | 9.65E-16 | 102 | PREDICTED: glutamate-gated chloride channel, (LOC112057699), transcript variant X6 |  |
| XM_024098215.2 | 80.18 | 111 | 22 | 0 | 10853611 | 10853721 | 1064 | 1174 | 9.65E-16 | 102 | PREDICTED: glutamate-gated chloride channel, (LOC112057699), transcript variant X5 |  |
| XM_024098213.2 | 80.18 | 111 | 22 | 0 | 10853611 | 10853721 | 1070 | 1180 | 9.65E-16 | 102 | PREDICTED: glutamate-gated chloride channel, (LOC112057699), transcript variant X4 |  |
| XM_024098212.2 | 80.18 | 111 | 22 | 0 | 10853611 | 10853721 | 1067 | 1177 | 9.65E-16 | 102 | PREDICTED: glutamate-gated chloride channel, (LOC112057699), transcript variant X3 |  |
| XM_024098210.2 | 80.18 | 111 | 22 | 0 | 10853611 | 10853721 | 1156 | 1266 | 9.65E-16 | 102 | PREDICTED: glutamate-gated chloride channel, (LOC112057699), transcript variant X2 |  |
| XM_024098209.2 | 80.18 | 111 | 22 | 0 | 10853611 | 10853721 | 1070 | 1180 | 9.65E-16 | 102 | PREDICTED: glutamate-gated chloride channel, (LOC112057699), transcript variant X1 |  |
| OX359226.1 | 80.18 | 111 | 22 | 0 | 10853611 | 10853721 | 12308084 | 12307974 | 9.65E-16 | 102 | Bicyclus anynana genome assembly, chromosome: 22 |  |
| XM_027367309.1 | 81.25 | 112 | 21 | 0 | 10853620 | 10853731 | 394 | 505 | 2.27E-17 | 108 | PREDICTED: Penaeus vannamei glutamate-gated chloride channel-like |  |
| XM_027377726.1 | 80.165 | 121 | 24 | 0 | 10855572 | 10855692 | 853 | 973 | 1.86E-18 | 111 | PREDICTED: Penaeus vannamei glutamate-gated chloride channel-like (LOC113824954), mRNA |  |
| XM_027367309.1 | 82.178 | 101 | 18 | 0 | 10855581 | 10855681 | 682 | 782 | 9.65E-16 | 102 | PREDICTED: Penaeus vannamei glutamate-gated chloride channel-like |  |
| XM_027351827.1 | 82.576 | 132 | 20 | 2 | 10855581 | 10855709 | 1100 | 1231 | 6.94E-24 | 129 | PREDICTED: Penaeus vannamei glutamate-gated chloride channel-like (LOC113801023), mRNA |  |
| XM_024098218.2 | 81.25 | 96 | 18 | 0 | 10855584 | 10855679 | 1316 | 1411 | 5.00E-13 | 93.3 | PREDICTED: glutamate-gated chloride channel, (LOC112057699), transcript variant X11 |  |
| XM_024098217.2 | 81.25 | 96 | 18 | 0 | 10855584 | 10855679 | 1319 | 1414 | 5.00E-13 | 93.3 | PREDICTED: glutamate-gated chloride channel, (LOC112057699), transcript variant X10 |  |
| XM_024098216.2 | 81.25 | 96 | 18 | 0 | 10855584 | 10855679 | 1322 | 1417 | 5.00E-13 | 93.3 | PREDICTED: glutamate-gated chloride channel, (LOC112057699), transcript variant X9 |  |
| XM_052888342.1 | 81.25 | 96 | 18 | 0 | 10855584 | 10855679 | 1349 | 1444 | 5.00E-13 | 93.3 | PREDICTED: glutamate-gated chloride channel, (LOC112057699), transcript variant X8 |  |
| XM_024098214.2 | 81.25 | 96 | 18 | 0 | 10855584 | 10855679 | 1352 | 1447 | 5.00E-13 | 93.3 | PREDICTED: glutamate-gated chloride channel, (LOC112057699), transcript variant X7 |  |
| XM_052888341.1 | 81.25 | 96 | 18 | 0 | 10855584 | 10855679 | 1352 | 1447 | 5.00E-13 | 93.3 | PREDICTED: glutamate-gated chloride channel, (LOC112057699), transcript variant X6 |  |
| XM_024098215.2 | 81.25 | 96 | 18 | 0 | 10855584 | 10855679 | 1352 | 1447 | 5.00E-13 | 93.3 | PREDICTED: glutamate-gated chloride channel, (LOC112057699), transcript variant X5 |  |
| XM_024098213.2 | 81.25 | 96 | 18 | 0 | 10855584 | 10855679 | 1355 | 1450 | 5.00E-13 | 93.3 | PREDICTED: glutamate-gated chloride channel, (LOC112057699), transcript variant X4 |  |
| XM_024098212.2 | 81.25 | 96 | 18 | 0 | 10855584 | 10855679 | 1355 | 1450 | 5.00E-13 | 93.3 | PREDICTED: glutamate-gated chloride channel, (LOC112057699), transcript variant X3 |  |
| XM_024098210.2 | 81.25 | 96 | 18 | 0 | 10855584 | 10855679 | 1444 | 1539 | 5.00E-13 | 93.3 | PREDICTED: glutamate-gated chloride channel, (LOC112057699), transcript variant X2 |  |
| XM_024098209.2 | 81.25 | 96 | 18 | 0 | 10855584 | 10855679 | 1358 | 1453 | 5.00E-13 | 93.3 | PREDICTED: glutamate-gated chloride channel, (LOC112057699), transcript variant X1 |  |
| OX359226.1 | 79.31 | 116 | 24 | 0 | 10855584 | 10855699 | 12306288 | 12306173 | 9.65E-16 | 102 | Bicyclus anynana genome assembly, chromosome: 22 |  |
| XM_027361146.1 | 77.606 | 259 | 56 | 1 | 10898322 | 10898580 | 856 | 600 | 3.84E-46 | 204 | PREDICTED: Penaeus vannamei 40S ribosomal protein S4-like |  |
| XM_027361146.1 | 77.606 | 259 | 56 | 1 | 10898322 | 10898580 | 983 | 1239 | 3.84E-46 | 204 | PREDICTED: Penaeus vannamei 40S ribosomal protein S4-like |  |
| OX359211.1 | 75.758 | 264 | 62 | 1 | 10898323 | 10898586 | 18592154 | 18591893 | 1.03E-40 | 186 | [Mus musculus genome assembly, chromosome: 17](https://www.ncbi.nlm.nih.gov/nuccore/OX389811.1) |  |
| XM_052882587.1 | 75.581 | 258 | 61 | 1 | 10898323 | 10898580 | 150 | 405 | 4.38E-39 | 179 | PREDICTED: Bicyclus anynana 40S ribosomal protein S4 (LOC112049427), transcript variant X2 |  |
| XM_052882586.1 | 75.581 | 258 | 61 | 1 | 10898323 | 10898580 | 149 | 404 | 4.38E-39 | 179 | PREDICTED: Bicyclus anynana 40S ribosomal protein S4 (LOC112049427), transcript variant X1 |  |
| CP122171.1 | 74.349 | 269 | 67 | 1 | 10898323 | 10898591 | 13042113 | 13042379 | 6.50E-37 | 172 | Drosophila melanogaster isolate dmeE_34_F0 chromosome 3L |  |
| CP023337.1 | 73.978 | 269 | 68 | 1 | 10898323 | 10898591 | 13035165 | 13035431 | 2.76E-35 | 168 | Drosophila melanogaster strain rover (forR) chromosome 3L |  |
| CP023331.1 | 73.978 | 269 | 68 | 1 | 10898323 | 10898591 | 13035204 | 13035470 | 2.76E-35 | 168 | Drosophila melanogaster strain sitter (fors) chromosome 3L |  |
| CP121945.1 | 73.978 | 269 | 68 | 1 | 10898323 | 10898591 | 13042113 | 13042379 | 2.76E-35 | 168 | Drosophila melanogaster isolate dmeA_05_F0 chromosome 3L |  |
| CP121927.1 | 73.978 | 269 | 68 | 1 | 10898323 | 10898591 | 13042113 | 13042379 | 2.76E-35 | 168 | Drosophila melanogaster isolate dmeA_01_F0 chromosome 3L |  |
| CP121921.1 | 73.978 | 269 | 68 | 1 | 10898323 | 10898591 | 13042113 | 13042379 | 2.76E-35 | 168 | Drosophila melanogaster isolate dmeA_01_F0 chromosome 3L |  |
| CP121933.1 | 73.978 | 269 | 68 | 1 | 10898323 | 10898591 | 13042113 | 13042379 | 2.76E-35 | 168 | Drosophila melanogaster isolate dmeA_01_M0 chromosome 3L |  |
| CP121939.1 | 73.978 | 269 | 68 | 1 | 10898323 | 10898591 | 13042113 | 13042379 | 2.76E-35 | 168 | Drosophila melanogaster isolate dmeA_05_F0 chromosome 3L |  |
| CP121968.1 | 73.978 | 269 | 68 | 1 | 10898323 | 10898591 | 13042113 | 13042379 | 2.76E-35 | 168 | Drosophila melanogaster isolate dmeA_15_F0 chromosome 3L |  |
| CP121951.1 | 73.978 | 269 | 68 | 1 | 10898323 | 10898591 | 13042113 | 13042379 | 2.76E-35 | 168 | Drosophila melanogaster isolate dmeA_05_M0 chromosome 3L |  |
| CP121962.1 | 73.978 | 269 | 68 | 1 | 10898323 | 10898591 | 13042113 | 13042379 | 2.76E-35 | 168 | Drosophila melanogaster isolate dmeA_15_F0 chromosome 3L |  |
| CP121974.1 | 73.978 | 269 | 68 | 1 | 10898323 | 10898591 | 13042113 | 13042379 | 2.76E-35 | 168 | Drosophila melanogaster isolate dmeA_15_M0 chromosome 3L |  |
| CP121997.1 | 73.978 | 269 | 68 | 1 | 10898323 | 10898591 | 13042113 | 13042379 | 2.76E-35 | 168 | Drosophila melanogaster isolate dmeA_18_F0 chromosome 3L |  |
| CP122003.1 | 73.978 | 269 | 68 | 1 | 10898323 | 10898591 | 13042113 | 13042379 | 2.76E-35 | 168 | Drosophila melanogaster isolate dmeA_18_M0 chromosome 3L |  |
| CP121986.1 | 73.978 | 269 | 68 | 1 | 10898323 | 10898591 | 13042113 | 13042379 | 2.76E-35 | 168 | Drosophila melanogaster isolate dmeA_18_F0 chromosome 3L |  |
| CP122075.1 | 73.978 | 269 | 68 | 1 | 10898323 | 10898591 | 13042113 | 13042379 | 2.76E-35 | 168 | Drosophila melanogaster isolate dmeE_27_M0 chromosome 3L |  |
| CP122015.1 | 73.978 | 269 | 68 | 1 | 10898323 | 10898591 | 13042113 | 13042379 | 2.76E-35 | 168 | Drosophila melanogaster isolate dmeA_23_F0 chromosome 3L |  |
| CP122021.1 | 73.978 | 269 | 68 | 1 | 10898323 | 10898591 | 13042113 | 13042379 | 2.76E-35 | 168 | Drosophila melanogaster isolate dmeA_23_F0 chromosome 3L |  |
| CP122045.1 | 73.978 | 269 | 68 | 1 | 10898323 | 10898591 | 13042113 | 13042379 | 2.76E-35 | 168 | Drosophila melanogaster isolate dmeA_25_F0 chromosome 3L |  |
| CP122039.1 | 73.978 | 269 | 68 | 1 | 10898323 | 10898591 | 13042113 | 13042379 | 2.76E-35 | 168 | Drosophila melanogaster isolate dmeA_25_F0 chromosome 3L |  |
| CP122051.1 | 73.978 | 269 | 68 | 1 | 10898323 | 10898591 | 13042113 | 13042379 | 2.76E-35 | 168 | Drosophila melanogaster isolate dmeA_25_M0 chromosome 3L |  |
| CP122069.1 | 73.978 | 269 | 68 | 1 | 10898323 | 10898591 | 13042113 | 13042379 | 2.76E-35 | 168 | Drosophila melanogaster isolate dmeE_27_F0 chromosome 3L |  |
| CP122063.1 | 73.978 | 269 | 68 | 1 | 10898323 | 10898591 | 13042113 | 13042379 | 2.76E-35 | 168 | Drosophila melanogaster isolate dmeE_27_F0 chromosome 3L |  |
| CP122093.1 | 73.978 | 269 | 68 | 1 | 10898323 | 10898591 | 13042113 | 13042379 | 2.76E-35 | 168 | Drosophila melanogaster isolate dmeE_28_F0 chromosome 3L |  |
| CP122087.1 | 73.978 | 269 | 68 | 1 | 10898323 | 10898591 | 13042113 | 13042379 | 2.76E-35 | 168 | Drosophila melanogaster isolate dmeE_28_F0 chromosome 3L |  |
| CP122099.1 | 73.978 | 269 | 68 | 1 | 10898323 | 10898591 | 13042113 | 13042379 | 2.76E-35 | 168 | Drosophila melanogaster isolate dmeE_28_M0 chromosome 3L |  |
| CP122111.1 | 73.978 | 269 | 68 | 1 | 10898323 | 10898591 | 13042113 | 13042379 | 2.76E-35 | 168 | Drosophila melanogaster isolate dmeE_29_F0 chromosome 3L |  |
| CP122117.1 | 73.978 | 269 | 68 | 1 | 10898323 | 10898591 | 13042113 | 13042379 | 2.76E-35 | 168 | Drosophila melanogaster isolate dmeE_29_F0 chromosome 3L |  |
| CP122123.1 | 73.978 | 269 | 68 | 1 | 10898323 | 10898591 | 13042113 | 13042379 | 2.76E-35 | 168 | Drosophila melanogaster isolate dmeE_29_M0 chromosome 3L |  |
| CP122177.1 | 73.978 | 269 | 68 | 1 | 10898323 | 10898591 | 13042113 | 13042379 | 2.76E-35 | 168 | Drosophila melanogaster isolate dmeE_34_M0 chromosome 3L |  |
| CP122129.1 | 73.978 | 269 | 68 | 1 | 10898323 | 10898591 | 13042113 | 13042379 | 2.76E-35 | 168 | Drosophila melanogaster isolate dmeE_30_F0 chromosome 3L |  |
| CP122135.1 | 73.978 | 269 | 68 | 1 | 10898323 | 10898591 | 13042113 | 13042379 | 2.76E-35 | 168 | Drosophila melanogaster isolate dmeE_30_F0 chromosome 3L |  |
| CP122141.1 | 73.978 | 269 | 68 | 1 | 10898323 | 10898591 | 13042113 | 13042379 | 2.76E-35 | 168 | Drosophila melanogaster isolate dmeE_30_M0 chromosome 3L |  |
| CP122147.1 | 73.978 | 269 | 68 | 1 | 10898323 | 10898591 | 13042113 | 13042379 | 2.76E-35 | 168 | Drosophila melanogaster isolate dmeE_32_F0 chromosome 3L |  |
| CP122153.1 | 73.978 | 269 | 68 | 1 | 10898323 | 10898591 | 13042113 | 13042379 | 2.76E-35 | 168 | Drosophila melanogaster isolate dmeE_32_F0 chromosome 3L |  |
| CP122159.1 | 73.978 | 269 | 68 | 1 | 10898323 | 10898591 | 13042113 | 13042379 | 2.76E-35 | 168 | Drosophila melanogaster isolate dmeE_32_M0 chromosome 3L |  |
| CP122165.1 | 73.978 | 269 | 68 | 1 | 10898323 | 10898591 | 13042113 | 13042379 | 2.76E-35 | 168 | Drosophila melanogaster isolate dmeE_34_F0 chromosome 3L |  |
| CP121992.1 | 73.978 | 269 | 68 | 1 | 10898323 | 10898591 | 13042113 | 13042379 | 2.76E-35 | 168 | Drosophila melanogaster isolate dmeA_05_M0 chromosome 3L |  |
| CP122254.1 | 73.978 | 269 | 68 | 1 | 10898323 | 10898591 | 13042113 | 13042379 | 2.76E-35 | 168 | Drosophila melanogaster isolate dmeE_30_M0 chromosome 3L |  |
| CP122259.1 | 73.978 | 269 | 68 | 1 | 10898323 | 10898591 | 13042113 | 13042379 | 2.76E-35 | 168 | Drosophila melanogaster isolate dmeE_32_M0 chromosome 3L |  |
| CP122264.1 | 73.978 | 269 | 68 | 1 | 10898323 | 10898591 | 13042113 | 13042379 | 2.76E-35 | 168 | Drosophila melanogaster isolate dmeE_34_M0 chromosome 3L |  |
| CP122249.1 | 73.978 | 269 | 68 | 1 | 10898323 | 10898591 | 13042113 | 13042379 | 2.76E-35 | 168 | Drosophila melanogaster isolate dmeE_29_M0 chromosome 3L |  |
| CP122033.1 | 73.978 | 269 | 68 | 1 | 10898323 | 10898591 | 13042113 | 13042379 | 2.76E-35 | 168 | Drosophila melanogaster isolate dmeE_29_M0 chromosome 3L |  |
| CP122105.1 | 73.978 | 269 | 68 | 1 | 10898323 | 10898591 | 13042113 | 13042379 | 2.76E-35 | 168 | Drosophila melanogaster isolate dmeE_28_M0 chromosome 3L |  |
| CP122081.1 | 73.978 | 269 | 68 | 1 | 10898323 | 10898591 | 13042113 | 13042379 | 2.76E-35 | 168 | Drosophila melanogaster isolate dmeE_27_M0 chromosome 3L |  |
| CP122057.1 | 73.978 | 269 | 68 | 1 | 10898323 | 10898591 | 13042113 | 13042379 | 2.76E-35 | 168 | Drosophila melanogaster isolate dmeA_25_M0 chromosome 3L |  |
| AE014296.5 | 73.978 | 269 | 68 | 1 | 10898323 | 10898591 | 13042113 | 13042379 | 2.76E-35 | 168 | Drosophila melanogaster chromosome 3L |  |
| AC093546.2 | 73.978 | 269 | 68 | 1 | 10898323 | 10898591 | 140303 | 140569 | 2.76E-35 | 168 | Drosophila melanogaster 3L BAC RP98-8G7 (Roswell Park Cancer Institute Drosophila BAC Library) complete sequence |  |
| CP122027.1 | 73.606 | 269 | 69 | 1 | 10898323 | 10898591 | 13042113 | 13042379 | 3.37E-34 | 163 | Drosophila melanogaster isolate dmeA_23_M0 chromosome 3L |  |
| CP121957.1 | 73.606 | 269 | 69 | 1 | 10898323 | 10898591 | 13042113 | 13042379 | 3.37E-34 | 163 | Drosophila melanogaster isolate dmeA_01_M0 chromosome 3L |  |
| CP122009.1 | 73.606 | 269 | 69 | 1 | 10898323 | 10898591 | 13042113 | 13042379 | 3.37E-34 | 163 | Drosophila melanogaster isolate dmeA_18_M0 chromosome 3L |  |
| CP121980.1 | 73.606 | 269 | 69 | 1 | 10898323 | 10898591 | 13042113 | 13042379 | 3.37E-34 | 163 | Drosophila melanogaster isolate dmeA_15_M0 chromosome 3L |  |
| NM_001300126.1 | 73.643 | 258 | 66 | 1 | 10898323 | 10898580 | 119 | 374 | 4.99E-32 | 157 | Dmel ribosomal protein S4 |  |
| NM_079329.3 | 73.643 | 258 | 66 | 1 | 10898323 | 10898580 | 218 | 473 | 4.99E-32 | 157 | Drosophila melanogaster ribosomal protein S4, transcript variant B |  |
| NM_168537.2 | 73.643 | 258 | 66 | 1 | 10898323 | 10898580 | 119 | 374 | 4.99E-32 | 157 | Drosophila melanogaster ribosomal protein S4, transcript variant A |  |
| BT011369.1 | 73.643 | 258 | 66 | 1 | 10898323 | 10898580 | 119 | 374 | 4.99E-32 | 157 | Drosophila melanogaster RE57333 full insert cDNA |  |
| CP122171.1 | 76.471 | 102 | 24 | 0 | 10903948 | 10904049 | 13042440 | 13042541 | 3.84E-08 | 77 | Drosophila melanogaster isolate dmeE_34_F0 chromosome 3L |  |
| CP023337.1 | 76.471 | 102 | 24 | 0 | 10903948 | 10904049 | 13035492 | 13035593 | 3.84E-08 | 77 | Drosophila melanogaster strain rover (forR) chromosome 3L |  |
| CP023331.1 | 76.471 | 102 | 24 | 0 | 10903948 | 10904049 | 13035531 | 13035632 | 3.84E-08 | 77 | Drosophila melanogaster strain sitter (fors) chromosome 3L |  |
| CP121945.1 | 76.471 | 102 | 24 | 0 | 10903948 | 10904049 | 13042440 | 13042541 | 3.84E-08 | 77 | Drosophila melanogaster isolate dmeA_05_F0 chromosome 3L |  |
| CP121927.1 | 76.471 | 102 | 24 | 0 | 10903948 | 10904049 | 13042440 | 13042541 | 3.84E-08 | 77 | Drosophila melanogaster isolate dmeA_01_F0 chromosome 3L |  |
| CP121921.1 | 76.471 | 102 | 24 | 0 | 10903948 | 10904049 | 13042440 | 13042541 | 3.84E-08 | 77 | Drosophila melanogaster isolate dmeA_01_F0 chromosome 3L |  |
| CP121933.1 | 76.471 | 102 | 24 | 0 | 10903948 | 10904049 | 13042440 | 13042541 | 3.84E-08 | 77 | Drosophila melanogaster isolate dmeA_01_M0 chromosome 3L |  |
| CP121939.1 | 76.471 | 102 | 24 | 0 | 10903948 | 10904049 | 13042440 | 13042541 | 3.84E-08 | 77 | Drosophila melanogaster isolate dmeA_05_F0 chromosome 3L |  |
| CP121968.1 | 76.471 | 102 | 24 | 0 | 10903948 | 10904049 | 13042440 | 13042541 | 3.84E-08 | 77 | Drosophila melanogaster isolate dmeA_15_F0 chromosome 3L |  |
| CP121951.1 | 76.471 | 102 | 24 | 0 | 10903948 | 10904049 | 13042440 | 13042541 | 3.84E-08 | 77 | Drosophila melanogaster isolate dmeA_05_M0 chromosome 3L |  |
| CP121962.1 | 76.471 | 102 | 24 | 0 | 10903948 | 10904049 | 13042440 | 13042541 | 3.84E-08 | 77 | Drosophila melanogaster isolate dmeA_15_F0 chromosome 3L |  |
| CP121974.1 | 76.471 | 102 | 24 | 0 | 10903948 | 10904049 | 13042440 | 13042541 | 3.84E-08 | 77 | Drosophila melanogaster isolate dmeA_15_M0 chromosome 3L |  |
| CP121997.1 | 76.471 | 102 | 24 | 0 | 10903948 | 10904049 | 13042440 | 13042541 | 3.84E-08 | 77 | Drosophila melanogaster isolate dmeA_18_F0 chromosome 3L |  |
| CP122003.1 | 76.471 | 102 | 24 | 0 | 10903948 | 10904049 | 13042440 | 13042541 | 3.84E-08 | 77 | Drosophila melanogaster isolate dmeA_18_M0 chromosome 3L |  |
| CP121986.1 | 76.471 | 102 | 24 | 0 | 10903948 | 10904049 | 13042440 | 13042541 | 3.84E-08 | 77 | Drosophila melanogaster isolate dmeA_18_F0 chromosome 3L |  |
| CP122075.1 | 76.471 | 102 | 24 | 0 | 10903948 | 10904049 | 13042440 | 13042541 | 3.84E-08 | 77 | Drosophila melanogaster isolate dmeE_27_M0 chromosome 3L |  |
| CP122015.1 | 76.471 | 102 | 24 | 0 | 10903948 | 10904049 | 13042440 | 13042541 | 3.84E-08 | 77 | Drosophila melanogaster isolate dmeA_23_F0 chromosome 3L |  |
| CP122021.1 | 76.471 | 102 | 24 | 0 | 10903948 | 10904049 | 13042440 | 13042541 | 3.84E-08 | 77 | Drosophila melanogaster isolate dmeA_23_F0 chromosome 3L |  |
| CP122045.1 | 76.471 | 102 | 24 | 0 | 10903948 | 10904049 | 13042440 | 13042541 | 3.84E-08 | 77 | Drosophila melanogaster isolate dmeA_25_F0 chromosome 3L |  |
| CP122039.1 | 76.471 | 102 | 24 | 0 | 10903948 | 10904049 | 13042440 | 13042541 | 3.84E-08 | 77 | Drosophila melanogaster isolate dmeA_25_F0 chromosome 3L |  |
| CP122051.1 | 76.471 | 102 | 24 | 0 | 10903948 | 10904049 | 13042440 | 13042541 | 3.84E-08 | 77 | Drosophila melanogaster isolate dmeA_25_M0 chromosome 3L |  |
| CP122069.1 | 76.471 | 102 | 24 | 0 | 10903948 | 10904049 | 13042440 | 13042541 | 3.84E-08 | 77 | Drosophila melanogaster isolate dmeE_27_F0 chromosome 3L |  |
| CP122063.1 | 76.471 | 102 | 24 | 0 | 10903948 | 10904049 | 13042440 | 13042541 | 3.84E-08 | 77 | Drosophila melanogaster isolate dmeE_27_F0 chromosome 3L |  |
| CP122093.1 | 76.471 | 102 | 24 | 0 | 10903948 | 10904049 | 13042440 | 13042541 | 3.84E-08 | 77 | Drosophila melanogaster isolate dmeE_28_F0 chromosome 3L |  |
| CP122087.1 | 76.471 | 102 | 24 | 0 | 10903948 | 10904049 | 13042440 | 13042541 | 3.84E-08 | 77 | Drosophila melanogaster isolate dmeE_28_F0 chromosome 3L |  |
| CP122099.1 | 76.471 | 102 | 24 | 0 | 10903948 | 10904049 | 13042440 | 13042541 | 3.84E-08 | 77 | Drosophila melanogaster isolate dmeE_28_M0 chromosome 3L |  |
| CP122111.1 | 76.471 | 102 | 24 | 0 | 10903948 | 10904049 | 13042440 | 13042541 | 3.84E-08 | 77 | Drosophila melanogaster isolate dmeE_29_F0 chromosome 3L |  |
| CP122117.1 | 76.471 | 102 | 24 | 0 | 10903948 | 10904049 | 13042440 | 13042541 | 3.84E-08 | 77 | Drosophila melanogaster isolate dmeE_29_F0 chromosome 3L |  |
| CP122123.1 | 76.471 | 102 | 24 | 0 | 10903948 | 10904049 | 13042440 | 13042541 | 3.84E-08 | 77 | Drosophila melanogaster isolate dmeE_29_M0 chromosome 3L |  |
| CP122177.1 | 76.471 | 102 | 24 | 0 | 10903948 | 10904049 | 13042440 | 13042541 | 3.84E-08 | 77 | Drosophila melanogaster isolate dmeE_34_M0 chromosome 3L |  |
| CP122129.1 | 76.471 | 102 | 24 | 0 | 10903948 | 10904049 | 13042440 | 13042541 | 3.84E-08 | 77 | Drosophila melanogaster isolate dmeE_30_F0 chromosome 3L |  |
| CP122135.1 | 76.471 | 102 | 24 | 0 | 10903948 | 10904049 | 13042440 | 13042541 | 3.84E-08 | 77 | Drosophila melanogaster isolate dmeE_30_F0 chromosome 3L |  |
| CP122141.1 | 76.471 | 102 | 24 | 0 | 10903948 | 10904049 | 13042440 | 13042541 | 3.84E-08 | 77 | Drosophila melanogaster isolate dmeE_30_M0 chromosome 3L |  |
| CP122147.1 | 76.471 | 102 | 24 | 0 | 10903948 | 10904049 | 13042440 | 13042541 | 3.84E-08 | 77 | Drosophila melanogaster isolate dmeE_32_F0 chromosome 3L |  |
| CP122153.1 | 76.471 | 102 | 24 | 0 | 10903948 | 10904049 | 13042440 | 13042541 | 3.84E-08 | 77 | Drosophila melanogaster isolate dmeE_32_F0 chromosome 3L |  |
| CP122159.1 | 76.471 | 102 | 24 | 0 | 10903948 | 10904049 | 13042440 | 13042541 | 3.84E-08 | 77 | Drosophila melanogaster isolate dmeE_32_M0 chromosome 3L |  |
| CP122165.1 | 76.471 | 102 | 24 | 0 | 10903948 | 10904049 | 13042440 | 13042541 | 3.84E-08 | 77 | Drosophila melanogaster isolate dmeE_34_F0 chromosome 3L |  |
| CP121992.1 | 76.471 | 102 | 24 | 0 | 10903948 | 10904049 | 13042440 | 13042541 | 3.84E-08 | 77 | Drosophila melanogaster isolate dmeA_05_M0 chromosome 3L |  |
| CP122254.1 | 76.471 | 102 | 24 | 0 | 10903948 | 10904049 | 13042440 | 13042541 | 3.84E-08 | 77 | Drosophila melanogaster isolate dmeE_30_M0 chromosome 3L |  |
| CP122259.1 | 76.471 | 102 | 24 | 0 | 10903948 | 10904049 | 13042440 | 13042541 | 3.84E-08 | 77 | Drosophila melanogaster isolate dmeE_32_M0 chromosome 3L |  |
| CP122264.1 | 76.471 | 102 | 24 | 0 | 10903948 | 10904049 | 13042440 | 13042541 | 3.84E-08 | 77 | Drosophila melanogaster isolate dmeE_34_M0 chromosome 3L |  |
| CP122249.1 | 76.471 | 102 | 24 | 0 | 10903948 | 10904049 | 13042440 | 13042541 | 3.84E-08 | 77 | Drosophila melanogaster isolate dmeE_29_M0 chromosome 3L |  |
| CP122033.1 | 76.471 | 102 | 24 | 0 | 10903948 | 10904049 | 13042440 | 13042541 | 3.84E-08 | 77 | Drosophila melanogaster isolate dmeE_29_M0 chromosome 3L |  |
| CP122105.1 | 76.471 | 102 | 24 | 0 | 10903948 | 10904049 | 13042440 | 13042541 | 3.84E-08 | 77 | Drosophila melanogaster isolate dmeE_28_M0 chromosome 3L |  |
| CP122081.1 | 76.471 | 102 | 24 | 0 | 10903948 | 10904049 | 13042440 | 13042541 | 3.84E-08 | 77 | Drosophila melanogaster isolate dmeE_27_M0 chromosome 3L |  |
| CP122057.1 | 76.471 | 102 | 24 | 0 | 10903948 | 10904049 | 13042440 | 13042541 | 3.84E-08 | 77 | Drosophila melanogaster isolate dmeA_25_M0 chromosome 3L |  |
| AE014296.5 | 76.471 | 102 | 24 | 0 | 10903948 | 10904049 | 13042440 | 13042541 | 3.84E-08 | 77 | Drosophila melanogaster chromosome 3L |  |
| AC093546.2 | 76.471 | 102 | 24 | 0 | 10903948 | 10904049 | 140630 | 140731 | 3.84E-08 | 77 | Drosophila melanogaster 3L BAC RP98-8G7 (Roswell Park Cancer Institute Drosophila BAC Library) complete sequence |  |
| CP122027.1 | 76.471 | 102 | 24 | 0 | 10903948 | 10904049 | 13042440 | 13042541 | 3.84E-08 | 77 | Drosophila melanogaster isolate dmeA_23_M0 chromosome 3L |  |
| CP121957.1 | 76.471 | 102 | 24 | 0 | 10903948 | 10904049 | 13042440 | 13042541 | 3.84E-08 | 77 | Drosophila melanogaster isolate dmeA_01_M0 chromosome 3L |  |
| CP122009.1 | 76.471 | 102 | 24 | 0 | 10903948 | 10904049 | 13042440 | 13042541 | 3.84E-08 | 77 | Drosophila melanogaster isolate dmeA_18_M0 chromosome 3L |  |
| CP121980.1 | 76.471 | 102 | 24 | 0 | 10903948 | 10904049 | 13042440 | 13042541 | 3.84E-08 | 77 | Drosophila melanogaster isolate dmeA_15_M0 chromosome 3L |  |
| XM_027361146.1 | 84.848 | 99 | 15 | 0 | 10903949 | 10904047 | 1239 | 1337 | 1.86E-18 | 112 | PREDICTED: Penaeus vannamei 40S ribosomal protein S4-like |  |
| XM_027361146.1 | 88 | 75 | 9 | 0 | 10903949 | 10904023 | 600 | 526 | 1.43E-13 | 96 | PREDICTED: Penaeus vannamei 40S ribosomal protein S4-like |  |
| NM_001300126.1 | 75.758 | 99 | 24 | 0 | 10903949 | 10904047 | 374 | 472 | 1.63E-06 | 71.6 | Dmel ribosomal protein S4 |  |
| NM_079329.3 | 75.758 | 99 | 24 | 0 | 10903949 | 10904047 | 473 | 571 | 1.63E-06 | 71.6 | Drosophila melanogaster ribosomal protein S4, transcript variant B |  |
| NM_168537.2 | 75.758 | 99 | 24 | 0 | 10903949 | 10904047 | 374 | 472 | 1.63E-06 | 71.6 | Drosophila melanogaster ribosomal protein S4, transcript variant A |  |
| BT011369.1 | 75.758 | 99 | 24 | 0 | 10903949 | 10904047 | 374 | 472 | 1.63E-06 | 71.6 | Drosophila melanogaster RE57333 full insert cDNA |  |
| XM_027361146.1 | 83.333 | 78 | 13 | 0 | 10903970 | 10904047 | 522 | 445 | 9.04E-10 | 83.3 | PREDICTED: Penaeus vannamei 40S ribosomal protein S4-like |  |
| CP122171.1 | 74.033 | 181 | 45 | 2 | 10905318 | 10905497 | 13043163 | 13043342 | 2.27E-17 | 108 | Drosophila melanogaster isolate dmeE_34_F0 chromosome 3L |  |
| CP023337.1 | 73.481 | 181 | 46 | 2 | 10905318 | 10905497 | 13036215 | 13036394 | 2.76E-16 | 104 | Drosophila melanogaster strain rover (forR) chromosome 3L |  |
| CP023331.1 | 73.481 | 181 | 46 | 2 | 10905318 | 10905497 | 13036254 | 13036433 | 2.76E-16 | 104 | Drosophila melanogaster strain sitter (fors) chromosome 3L |  |
| CP121945.1 | 72.928 | 181 | 47 | 2 | 10905318 | 10905497 | 13043163 | 13043342 | 1.18E-14 | 99.6 | Drosophila melanogaster isolate dmeA_05_F0 chromosome 3L |  |
| CP121927.1 | 74.033 | 181 | 45 | 2 | 10905318 | 10905497 | 13043163 | 13043342 | 2.27E-17 | 108 | Drosophila melanogaster isolate dmeA_01_F0 chromosome 3L |  |
| CP121921.1 | 73.481 | 181 | 46 | 2 | 10905318 | 10905497 | 13043163 | 13043342 | 2.76E-16 | 104 | Drosophila melanogaster isolate dmeA_01_F0 chromosome 3L |  |
| CP121933.1 | 73.481 | 181 | 46 | 2 | 10905318 | 10905497 | 13043163 | 13043342 | 2.76E-16 | 104 | Drosophila melanogaster isolate dmeA_01_M0 chromosome 3L |  |
| CP121939.1 | 73.481 | 181 | 46 | 2 | 10905318 | 10905497 | 13043163 | 13043342 | 2.76E-16 | 104 | Drosophila melanogaster isolate dmeA_05_F0 chromosome 3L |  |
| CP121968.1 | 73.481 | 181 | 46 | 2 | 10905318 | 10905497 | 13043163 | 13043342 | 2.76E-16 | 104 | Drosophila melanogaster isolate dmeA_15_F0 chromosome 3L |  |
| CP121951.1 | 73.481 | 181 | 46 | 2 | 10905318 | 10905497 | 13043163 | 13043342 | 2.76E-16 | 104 | Drosophila melanogaster isolate dmeA_05_M0 chromosome 3L |  |
| CP121962.1 | 73.481 | 181 | 46 | 2 | 10905318 | 10905497 | 13043163 | 13043342 | 2.76E-16 | 104 | Drosophila melanogaster isolate dmeA_15_F0 chromosome 3L |  |
| CP121974.1 | 73.481 | 181 | 46 | 2 | 10905318 | 10905497 | 13043163 | 13043342 | 2.76E-16 | 104 | Drosophila melanogaster isolate dmeA_15_M0 chromosome 3L |  |
| CP121997.1 | 73.481 | 181 | 46 | 2 | 10905318 | 10905497 | 13043163 | 13043342 | 2.76E-16 | 104 | Drosophila melanogaster isolate dmeA_18_F0 chromosome 3L |  |
| CP122003.1 | 73.481 | 181 | 46 | 2 | 10905318 | 10905497 | 13043163 | 13043342 | 2.76E-16 | 104 | Drosophila melanogaster isolate dmeA_18_M0 chromosome 3L |  |
| CP121986.1 | 73.481 | 181 | 46 | 2 | 10905318 | 10905497 | 13043163 | 13043342 | 2.76E-16 | 104 | Drosophila melanogaster isolate dmeA_18_F0 chromosome 3L |  |
| CP122075.1 | 73.481 | 181 | 46 | 2 | 10905318 | 10905497 | 13043163 | 13043342 | 2.76E-16 | 104 | Drosophila melanogaster isolate dmeE_27_M0 chromosome 3L |  |
| CP122015.1 | 73.481 | 181 | 46 | 2 | 10905318 | 10905497 | 13043163 | 13043342 | 2.76E-16 | 104 | Drosophila melanogaster isolate dmeA_23_F0 chromosome 3L |  |
| CP122021.1 | 74.033 | 181 | 45 | 2 | 10905318 | 10905497 | 13043163 | 13043342 | 2.27E-17 | 108 | Drosophila melanogaster isolate dmeA_23_F0 chromosome 3L |  |
| CP122045.1 | 73.481 | 181 | 46 | 2 | 10905318 | 10905497 | 13043163 | 13043342 | 2.76E-16 | 104 | Drosophila melanogaster isolate dmeA_25_F0 chromosome 3L |  |
| CP122039.1 | 74.033 | 181 | 45 | 2 | 10905318 | 10905497 | 13043163 | 13043342 | 2.27E-17 | 108 | Drosophila melanogaster isolate dmeA_25_F0 chromosome 3L |  |
| CP122051.1 | 73.481 | 181 | 46 | 2 | 10905318 | 10905497 | 13043163 | 13043342 | 2.76E-16 | 104 | Drosophila melanogaster isolate dmeA_25_M0 chromosome 3L |  |
| CP122069.1 | 73.481 | 181 | 46 | 2 | 10905318 | 10905497 | 13043163 | 13043342 | 2.76E-16 | 104 | Drosophila melanogaster isolate dmeE_27_F0 chromosome 3L |  |
| CP122063.1 | 73.481 | 181 | 46 | 2 | 10905318 | 10905497 | 13043163 | 13043342 | 2.76E-16 | 104 | Drosophila melanogaster isolate dmeE_27_F0 chromosome 3L |  |
| CP122093.1 | 73.481 | 181 | 46 | 2 | 10905318 | 10905497 | 13043163 | 13043342 | 2.76E-16 | 104 | Drosophila melanogaster isolate dmeE_28_F0 chromosome 3L |  |
| CP122087.1 | 73.481 | 181 | 46 | 2 | 10905318 | 10905497 | 13043163 | 13043342 | 2.76E-16 | 104 | Drosophila melanogaster isolate dmeE_28_F0 chromosome 3L |  |
| CP122099.1 | 73.481 | 181 | 46 | 2 | 10905318 | 10905497 | 13043163 | 13043342 | 2.76E-16 | 104 | Drosophila melanogaster isolate dmeE_28_M0 chromosome 3L |  |
| CP122111.1 | 72.928 | 181 | 47 | 2 | 10905318 | 10905497 | 13043163 | 13043342 | 1.18E-14 | 99.6 | Drosophila melanogaster isolate dmeE_29_F0 chromosome 3L |  |
| CP122117.1 | 73.481 | 181 | 46 | 2 | 10905318 | 10905497 | 13043163 | 13043342 | 2.76E-16 | 104 | Drosophila melanogaster isolate dmeE_29_F0 chromosome 3L |  |
| CP122123.1 | 73.481 | 181 | 46 | 2 | 10905318 | 10905497 | 13043163 | 13043342 | 2.76E-16 | 104 | Drosophila melanogaster isolate dmeE_29_M0 chromosome 3L |  |
| CP122177.1 | 73.481 | 181 | 46 | 2 | 10905318 | 10905497 | 13043163 | 13043342 | 2.76E-16 | 104 | Drosophila melanogaster isolate dmeE_34_M0 chromosome 3L |  |
| CP122129.1 | 73.481 | 181 | 46 | 2 | 10905318 | 10905497 | 13043163 | 13043342 | 2.76E-16 | 104 | Drosophila melanogaster isolate dmeE_30_F0 chromosome 3L |  |
| CP122135.1 | 73.481 | 181 | 46 | 2 | 10905318 | 10905497 | 13043163 | 13043342 | 2.76E-16 | 104 | Drosophila melanogaster isolate dmeE_30_F0 chromosome 3L |  |
| CP122141.1 | 73.481 | 181 | 46 | 2 | 10905318 | 10905497 | 13043163 | 13043342 | 2.76E-16 | 104 | Drosophila melanogaster isolate dmeE_30_M0 chromosome 3L |  |
| CP122147.1 | 73.481 | 181 | 46 | 2 | 10905318 | 10905497 | 13043163 | 13043342 | 2.76E-16 | 104 | Drosophila melanogaster isolate dmeE_32_F0 chromosome 3L |  |
| CP122153.1 | 74.033 | 181 | 45 | 2 | 10905318 | 10905497 | 13043163 | 13043342 | 2.27E-17 | 108 | Drosophila melanogaster isolate dmeE_32_F0 chromosome 3L |  |
| CP122159.1 | 73.481 | 181 | 46 | 2 | 10905318 | 10905497 | 13043163 | 13043342 | 2.76E-16 | 104 | Drosophila melanogaster isolate dmeE_32_M0 chromosome 3L |  |
| CP122165.1 | 73.481 | 181 | 46 | 2 | 10905318 | 10905497 | 13043163 | 13043342 | 2.76E-16 | 104 | Drosophila melanogaster isolate dmeE_34_F0 chromosome 3L |  |
| CP121992.1 | 73.481 | 181 | 46 | 2 | 10905318 | 10905497 | 13043163 | 13043342 | 2.76E-16 | 104 | Drosophila melanogaster isolate dmeA_05_M0 chromosome 3L |  |
| CP122254.1 | 73.481 | 181 | 46 | 2 | 10905318 | 10905497 | 13043163 | 13043342 | 2.76E-16 | 104 | Drosophila melanogaster isolate dmeE_30_M0 chromosome 3L |  |
| CP122259.1 | 73.481 | 181 | 46 | 2 | 10905318 | 10905497 | 13043163 | 13043342 | 2.76E-16 | 104 | Drosophila melanogaster isolate dmeE_32_M0 chromosome 3L |  |
| CP122264.1 | 73.481 | 181 | 46 | 2 | 10905318 | 10905497 | 13043163 | 13043342 | 2.76E-16 | 104 | Drosophila melanogaster isolate dmeE_34_M0 chromosome 3L |  |
| CP122249.1 | 73.481 | 181 | 46 | 2 | 10905318 | 10905497 | 13043163 | 13043342 | 2.76E-16 | 104 | Drosophila melanogaster isolate dmeE_29_M0 chromosome 3L |  |
| CP122033.1 | 73.481 | 181 | 46 | 2 | 10905318 | 10905497 | 13043163 | 13043342 | 2.76E-16 | 104 | Drosophila melanogaster isolate dmeE_29_M0 chromosome 3L |  |
| CP122105.1 | 74.033 | 181 | 45 | 2 | 10905318 | 10905497 | 13043163 | 13043342 | 2.27E-17 | 108 | Drosophila melanogaster isolate dmeE_28_M0 chromosome 3L |  |
| CP122081.1 | 73.481 | 181 | 46 | 2 | 10905318 | 10905497 | 13043163 | 13043342 | 2.76E-16 | 104 | Drosophila melanogaster isolate dmeE_27_M0 chromosome 3L |  |
| CP122057.1 | 73.481 | 181 | 46 | 2 | 10905318 | 10905497 | 13043163 | 13043342 | 2.76E-16 | 104 | Drosophila melanogaster isolate dmeA_25_M0 chromosome 3L |  |
| AE014296.5 | 73.481 | 181 | 46 | 2 | 10905318 | 10905497 | 13043163 | 13043342 | 2.76E-16 | 104 | Drosophila melanogaster chromosome 3L |  |
| AC093546.2 | 73.481 | 181 | 46 | 2 | 10905318 | 10905497 | 141353 | 141532 | 2.76E-16 | 104 | Drosophila melanogaster 3L BAC RP98-8G7 (Roswell Park Cancer Institute Drosophila BAC Library) complete sequence |  |
| CP122027.1 | 73.481 | 181 | 46 | 2 | 10905318 | 10905497 | 13043163 | 13043342 | 2.76E-16 | 104 | Drosophila melanogaster isolate dmeA_23_M0 chromosome 3L |  |
| CP121957.1 | 73.481 | 181 | 46 | 2 | 10905318 | 10905497 | 13043163 | 13043342 | 2.76E-16 | 104 | Drosophila melanogaster isolate dmeA_01_M0 chromosome 3L |  |
| CP122009.1 | 73.481 | 181 | 46 | 2 | 10905318 | 10905497 | 13043163 | 13043342 | 2.76E-16 | 104 | Drosophila melanogaster isolate dmeA_18_M0 chromosome 3L |  |
| CP121980.1 | 74.033 | 181 | 45 | 2 | 10905318 | 10905497 | 13043163 | 13043342 | 2.27E-17 | 108 | Drosophila melanogaster isolate dmeA_15_M0 chromosome 3L |  |
| XM_027361146.1 | 80.702 | 171 | 29 | 2 | 10905319 | 10905487 | 446 | 278 | 1.74E-31 | 155 | PREDICTED: Penaeus vannamei 40S ribosomal protein S4-like |  |
| XM_027361146.1 | 80.702 | 171 | 29 | 2 | 10905319 | 10905487 | 1336 | 1504 | 1.74E-31 | 155 | PREDICTED: Penaeus vannamei 40S ribosomal protein S4-like |  |
| XM_052882587.1 | 74.586 | 181 | 40 | 4 | 10905319 | 10905496 | 502 | 679 | 2.27E-17 | 107 | PREDICTED: Bicyclus anynana 40S ribosomal protein S4 (LOC112049427), transcript variant X2 |  |
| XM_052882586.1 | 74.586 | 181 | 40 | 4 | 10905319 | 10905496 | 501 | 678 | 2.27E-17 | 107 | PREDICTED: Bicyclus anynana 40S ribosomal protein S4 (LOC112049427), transcript variant X1 |  |
| NM_001300126.1 | 72.626 | 179 | 47 | 2 | 10905319 | 10905496 | 471 | 648 | 1.43E-13 | 96 | Dmel ribosomal protein S4 |  |
| NM_079329.3 | 72.626 | 179 | 47 | 2 | 10905319 | 10905496 | 570 | 747 | 1.43E-13 | 96 | Drosophila melanogaster ribosomal protein S4, transcript variant B |  |
| NM_168537.2 | 72.626 | 179 | 47 | 2 | 10905319 | 10905496 | 471 | 648 | 1.43E-13 | 96 | Drosophila melanogaster ribosomal protein S4, transcript variant A |  |
| BT011369.1 | 72.626 | 179 | 47 | 2 | 10905319 | 10905496 | 471 | 648 | 1.43E-13 | 96 | Drosophila melanogaster RE57333 full insert cDNA |  |
| NM_001300126.1 | 78.378 | 111 | 24 | 0 | 10907661 | 10907771 | 640 | 750 | 5.00E-13 | 93.3 | Dmel ribosomal protein S4 |  |
| NM_079329.3 | 78.378 | 111 | 24 | 0 | 10907661 | 10907771 | 739 | 849 | 5.00E-13 | 93.3 | Drosophila melanogaster ribosomal protein S4, transcript variant B |  |
| NM_168537.2 | 78.378 | 111 | 24 | 0 | 10907661 | 10907771 | 640 | 750 | 5.00E-13 | 93.3 | Drosophila melanogaster ribosomal protein S4, transcript variant A |  |
| BT011369.1 | 78.378 | 111 | 24 | 0 | 10907661 | 10907771 | 640 | 750 | 5.00E-13 | 93.3 | Drosophila melanogaster RE57333 full insert cDNA |  |
| OX359211.1 | 77.863 | 131 | 29 | 0 | 10907662 | 10907792 | 18588156 | 18588026 | 7.92E-17 | 106 | Bicyclus anynana genome assembly, chromosome: 7 |  |
| XM_052882587.1 | 77.863 | 131 | 29 | 0 | 10907662 | 10907792 | 672 | 802 | 7.92E-17 | 106 | PREDICTED: Bicyclus anynana 40S ribosomal protein S4 (LOC112049427), transcript variant X2 |  |
| XM_052882586.1 | 77.863 | 131 | 29 | 0 | 10907662 | 10907792 | 671 | 801 | 7.92E-17 | 106 | PREDICTED: Bicyclus anynana 40S ribosomal protein S4 (LOC112049427), transcript variant X1 |  |
| XM_027361146.1 | 81.481 | 135 | 25 | 0 | 10907663 | 10907797 | 275 | 141 | 1.99E-24 | 132 | PREDICTED: Penaeus vannamei 40S ribosomal protein S4-like |  |
| XM_027361146.1 | 81.481 | 135 | 25 | 0 | 10907663 | 10907797 | 1507 | 1641 | 1.99E-24 | 132 | PREDICTED: Penaeus vannamei 40S ribosomal protein S4-like |  |
| CP122171.1 | 78.899 | 109 | 23 | 0 | 10907663 | 10907771 | 13043571 | 13043679 | 5.00E-13 | 94.2 | Drosophila melanogaster isolate dmeE_34_F0 chromosome 3L |  |
| CP023337.1 | 78.899 | 109 | 23 | 0 | 10907663 | 10907771 | 13036623 | 13036731 | 5.00E-13 | 94.2 | Drosophila melanogaster strain rover (forR) chromosome 3L |  |
| CP023331.1 | 78.899 | 109 | 23 | 0 | 10907663 | 10907771 | 13036662 | 13036770 | 5.00E-13 | 94.2 | Drosophila melanogaster strain sitter (fors) chromosome 3L |  |
| CP121945.1 | 78.899 | 109 | 23 | 0 | 10907663 | 10907771 | 13043571 | 13043679 | 5.00E-13 | 94.2 | Drosophila melanogaster isolate dmeA_05_F0 chromosome 3L |  |
| CP121927.1 | 78.899 | 109 | 23 | 0 | 10907663 | 10907771 | 13043571 | 13043679 | 5.00E-13 | 94.2 | Drosophila melanogaster isolate dmeA_01_F0 chromosome 3L |  |
| CP121921.1 | 78.899 | 109 | 23 | 0 | 10907663 | 10907771 | 13043571 | 13043679 | 5.00E-13 | 94.2 | Drosophila melanogaster isolate dmeA_01_F0 chromosome 3L |  |
| CP121933.1 | 78.899 | 109 | 23 | 0 | 10907663 | 10907771 | 13043571 | 13043679 | 5.00E-13 | 94.2 | Drosophila melanogaster isolate dmeA_01_M0 chromosome 3L |  |
| CP121939.1 | 78.899 | 109 | 23 | 0 | 10907663 | 10907771 | 13043571 | 13043679 | 5.00E-13 | 94.2 | Drosophila melanogaster isolate dmeA_05_F0 chromosome 3L |  |
| CP121968.1 | 78.899 | 109 | 23 | 0 | 10907663 | 10907771 | 13043571 | 13043679 | 5.00E-13 | 94.2 | Drosophila melanogaster isolate dmeA_15_F0 chromosome 3L |  |
| CP121951.1 | 78.899 | 109 | 23 | 0 | 10907663 | 10907771 | 13043571 | 13043679 | 5.00E-13 | 94.2 | Drosophila melanogaster isolate dmeA_05_M0 chromosome 3L |  |
| CP121962.1 | 78.899 | 109 | 23 | 0 | 10907663 | 10907771 | 13043571 | 13043679 | 5.00E-13 | 94.2 | Drosophila melanogaster isolate dmeA_15_F0 chromosome 3L |  |
| CP121974.1 | 78.899 | 109 | 23 | 0 | 10907663 | 10907771 | 13043571 | 13043679 | 5.00E-13 | 94.2 | Drosophila melanogaster isolate dmeA_15_M0 chromosome 3L |  |
| CP121997.1 | 78.899 | 109 | 23 | 0 | 10907663 | 10907771 | 13043571 | 13043679 | 5.00E-13 | 94.2 | Drosophila melanogaster isolate dmeA_18_F0 chromosome 3L |  |
| CP122003.1 | 78.899 | 109 | 23 | 0 | 10907663 | 10907771 | 13043571 | 13043679 | 5.00E-13 | 94.2 | Drosophila melanogaster isolate dmeA_18_M0 chromosome 3L |  |
| CP121986.1 | 78.899 | 109 | 23 | 0 | 10907663 | 10907771 | 13043571 | 13043679 | 5.00E-13 | 94.2 | Drosophila melanogaster isolate dmeA_18_F0 chromosome 3L |  |
| CP122075.1 | 79.817 | 109 | 22 | 0 | 10907663 | 10907771 | 13043571 | 13043679 | 1.18E-14 | 98.7 | Drosophila melanogaster isolate dmeE_27_M0 chromosome 3L |  |
| CP122015.1 | 78.899 | 109 | 23 | 0 | 10907663 | 10907771 | 13043571 | 13043679 | 5.00E-13 | 94.2 | Drosophila melanogaster isolate dmeA_23_F0 chromosome 3L |  |
| CP122021.1 | 78.899 | 109 | 23 | 0 | 10907663 | 10907771 | 13043571 | 13043679 | 5.00E-13 | 94.2 | Drosophila melanogaster isolate dmeA_23_F0 chromosome 3L |  |
| CP122045.1 | 78.899 | 109 | 23 | 0 | 10907663 | 10907771 | 13043571 | 13043679 | 5.00E-13 | 94.2 | Drosophila melanogaster isolate dmeA_25_F0 chromosome 3L |  |
| CP122039.1 | 78.899 | 109 | 23 | 0 | 10907663 | 10907771 | 13043571 | 13043679 | 5.00E-13 | 94.2 | Drosophila melanogaster isolate dmeA_25_F0 chromosome 3L |  |
| CP122051.1 | 78.899 | 109 | 23 | 0 | 10907663 | 10907771 | 13043571 | 13043679 | 5.00E-13 | 94.2 | Drosophila melanogaster isolate dmeA_25_M0 chromosome 3L |  |
| CP122069.1 | 78.899 | 109 | 23 | 0 | 10907663 | 10907771 | 13043571 | 13043679 | 5.00E-13 | 94.2 | Drosophila melanogaster isolate dmeE_27_F0 chromosome 3L |  |
| CP122063.1 | 78.899 | 109 | 23 | 0 | 10907663 | 10907771 | 13043571 | 13043679 | 5.00E-13 | 94.2 | Drosophila melanogaster isolate dmeE_27_F0 chromosome 3L |  |
| CP122093.1 | 78.899 | 109 | 23 | 0 | 10907663 | 10907771 | 13043571 | 13043679 | 5.00E-13 | 94.2 | Drosophila melanogaster isolate dmeE_28_F0 chromosome 3L |  |
| CP122087.1 | 78.899 | 109 | 23 | 0 | 10907663 | 10907771 | 13043571 | 13043679 | 5.00E-13 | 94.2 | Drosophila melanogaster isolate dmeE_28_F0 chromosome 3L |  |
| CP122099.1 | 78.899 | 109 | 23 | 0 | 10907663 | 10907771 | 13043571 | 13043679 | 5.00E-13 | 94.2 | Drosophila melanogaster isolate dmeE_28_M0 chromosome 3L |  |
| CP122111.1 | 78.899 | 109 | 23 | 0 | 10907663 | 10907771 | 13043571 | 13043679 | 5.00E-13 | 94.2 | Drosophila melanogaster isolate dmeE_29_F0 chromosome 3L |  |
| CP122117.1 | 78.899 | 109 | 23 | 0 | 10907663 | 10907771 | 13043571 | 13043679 | 5.00E-13 | 94.2 | Drosophila melanogaster isolate dmeE_29_F0 chromosome 3L |  |
| CP122123.1 | 78.899 | 109 | 23 | 0 | 10907663 | 10907771 | 13043571 | 13043679 | 5.00E-13 | 94.2 | Drosophila melanogaster isolate dmeE_29_M0 chromosome 3L |  |
| CP122177.1 | 78.899 | 109 | 23 | 0 | 10907663 | 10907771 | 13043571 | 13043679 | 5.00E-13 | 94.2 | Drosophila melanogaster isolate dmeE_34_M0 chromosome 3L |  |
| CP122129.1 | 78.899 | 109 | 23 | 0 | 10907663 | 10907771 | 13043571 | 13043679 | 5.00E-13 | 94.2 | Drosophila melanogaster isolate dmeE_30_F0 chromosome 3L |  |
| CP122135.1 | 78.899 | 109 | 23 | 0 | 10907663 | 10907771 | 13043571 | 13043679 | 5.00E-13 | 94.2 | Drosophila melanogaster isolate dmeE_30_F0 chromosome 3L |  |
| CP122141.1 | 78.899 | 109 | 23 | 0 | 10907663 | 10907771 | 13043571 | 13043679 | 5.00E-13 | 94.2 | Drosophila melanogaster isolate dmeE_30_M0 chromosome 3L |  |
| CP122147.1 | 78.899 | 109 | 23 | 0 | 10907663 | 10907771 | 13043571 | 13043679 | 5.00E-13 | 94.2 | Drosophila melanogaster isolate dmeE_32_F0 chromosome 3L |  |
| CP122153.1 | 78.899 | 109 | 23 | 0 | 10907663 | 10907771 | 13043571 | 13043679 | 5.00E-13 | 94.2 | Drosophila melanogaster isolate dmeE_32_F0 chromosome 3L |  |
| CP122159.1 | 78.899 | 109 | 23 | 0 | 10907663 | 10907771 | 13043571 | 13043679 | 5.00E-13 | 94.2 | Drosophila melanogaster isolate dmeE_32_M0 chromosome 3L |  |
| CP122165.1 | 78.899 | 109 | 23 | 0 | 10907663 | 10907771 | 13043571 | 13043679 | 5.00E-13 | 94.2 | Drosophila melanogaster isolate dmeE_34_F0 chromosome 3L |  |
| CP121992.1 | 78.899 | 109 | 23 | 0 | 10907663 | 10907771 | 13043571 | 13043679 | 5.00E-13 | 94.2 | Drosophila melanogaster isolate dmeA_05_M0 chromosome 3L |  |
| CP122254.1 | 78.899 | 109 | 23 | 0 | 10907663 | 10907771 | 13043571 | 13043679 | 5.00E-13 | 94.2 | Drosophila melanogaster isolate dmeE_30_M0 chromosome 3L |  |
| CP122259.1 | 78.899 | 109 | 23 | 0 | 10907663 | 10907771 | 13043571 | 13043679 | 5.00E-13 | 94.2 | Drosophila melanogaster isolate dmeE_32_M0 chromosome 3L |  |
| CP122264.1 | 78.899 | 109 | 23 | 0 | 10907663 | 10907771 | 13043571 | 13043679 | 5.00E-13 | 94.2 | Drosophila melanogaster isolate dmeE_34_M0 chromosome 3L |  |
| CP122249.1 | 79.817 | 109 | 22 | 0 | 10907663 | 10907771 | 13043571 | 13043679 | 1.18E-14 | 98.7 | Drosophila melanogaster isolate dmeE_29_M0 chromosome 3L |  |
| CP122033.1 | 78.899 | 109 | 23 | 0 | 10907663 | 10907771 | 13043571 | 13043679 | 5.00E-13 | 94.2 | Drosophila melanogaster isolate dmeE_29_M0 chromosome 3L |  |
| CP122105.1 | 78.899 | 109 | 23 | 0 | 10907663 | 10907771 | 13043571 | 13043679 | 5.00E-13 | 94.2 | Drosophila melanogaster isolate dmeE_28_M0 chromosome 3L |  |
| CP122081.1 | 79.817 | 109 | 22 | 0 | 10907663 | 10907771 | 13043571 | 13043679 | 1.18E-14 | 98.7 | Drosophila melanogaster isolate dmeE_27_M0 chromosome 3L |  |
| CP122057.1 | 78.899 | 109 | 23 | 0 | 10907663 | 10907771 | 13043571 | 13043679 | 5.00E-13 | 94.2 | Drosophila melanogaster isolate dmeA_25_M0 chromosome 3L |  |
| AE014296.5 | 78.899 | 109 | 23 | 0 | 10907663 | 10907771 | 13043571 | 13043679 | 5.00E-13 | 94.2 | Drosophila melanogaster chromosome 3L |  |
| AC093546.2 | 78.899 | 109 | 23 | 0 | 10907663 | 10907771 | 141761 | 141869 | 5.00E-13 | 94.2 | Drosophila melanogaster 3L BAC RP98-8G7 (Roswell Park Cancer Institute Drosophila BAC Library) complete sequence |  |
| CP122027.1 | 78.899 | 109 | 23 | 0 | 10907663 | 10907771 | 13043571 | 13043679 | 5.00E-13 | 94.2 | Drosophila melanogaster isolate dmeA_23_M0 chromosome 3L |  |
| CP121957.1 | 78.899 | 109 | 23 | 0 | 10907663 | 10907771 | 13043571 | 13043679 | 5.00E-13 | 94.2 | Drosophila melanogaster isolate dmeA_01_M0 chromosome 3L |  |
| CP122009.1 | 80.734 | 109 | 21 | 0 | 10907663 | 10907771 | 13043571 | 13043679 | 9.65E-16 | 103 | Drosophila melanogaster isolate dmeA_18_M0 chromosome 3L |  |
| CP121980.1 | 78.899 | 109 | 23 | 0 | 10907663 | 10907771 | 13043571 | 13043679 | 5.00E-13 | 94.2 | Drosophila melanogaster isolate dmeA_15_M0 chromosome 3L |  |
| XM_027361146.1 | 81.89 | 127 | 23 | 0 | 10909211 | 10909337 | 145 | 19 | 8.46E-23 | 126 | PREDICTED: Penaeus vannamei 40S ribosomal protein S4-like |  |
| XM_027361146.1 | 81.89 | 127 | 23 | 0 | 10909211 | 10909337 | 1637 | 1763 | 8.46E-23 | 126 | PREDICTED: Penaeus vannamei 40S ribosomal protein S4-like |  |
